# Multi-objective optimization of total solid waste filling ratio based on NSGA-III and entropy-weighted TOPSIS method

**DOI:** 10.1038/s41598-026-55416-w

**Published:** 2026-06-06

**Authors:** Huisheng Qu, Tiantian Li, Lang Liu, Mengbo Zhu, Zhenmin Luo, Caixin Zhang, Chen Huang, Xin Cao, Mingyang Song

**Affiliations:** 1 College of Energy and Mining Engineering, Xi’an University of Science and Technology, Xi ’an, 710054 China; 2 Key Laboratory of Western Mines and Hazards Prevention, Ministry of Education of China, Xi’an, 710054 China

**Keywords:** Total solid waste, RSM-BBD, NSGA-III, Entropy-weighted TOPSIS, Hydration mechanism, Energy science and technology, Engineering, Environmental sciences, Materials science

## Abstract

To alleviate the growing disposal pressure of coal-based solid wastes and high-salinity wastewater from coal-chemical industries, and to reduce the cost and carbon footprint related to conventional Portland-cement binders for backfilling, the study develops a cemented backfill material derived entirely from industrial by-products. Modified magnesium slag (MMS), coarse coal gasification slag (CGS), desulfurized gypsum (DG), and high-salinity wastewater (HSW) were combined to formulate the total solid waste–based cemented backfill (TSW-CPB) system, and a multi-objective mix-design strategy integrating response surface methodology (RSM)–NSGA-III–entropy-weighted TOPSIS was established. Using an RSM-based Box–Behnken design (BBD), mass concentration (72–80%), aggregate-to-cement ratio (1–2), and aggregate grading index (0.3–0.7) were selected as input factors, while the unconfined compressive strength (UCS) at the 3rd, 14th, and 28th day and the slump were used as responses. The developed regression models were highly significant, without lack-of-fit (*P* < 0.0001; *P* > 0.05). Under workability constraints (slump of 15–20 cm for pumping and 23–27 cm for self-flowing) and with economic performance incorporated, a Pareto-optimum solution set was generated and further screened for each workability interval. For the optimal mix proportion under pumping condition, the mass concentration, aggregate-to-cement ratio, and grading index were separately 78.758%, 1.3911, and 0.30504, whereas for the self-flowing condition, they were 77.527%, 1.0003, and 0.5864, respectively. Hydration calorimetry and microstructural analyses indicate that CGS retards the reaction kinetics of the binary system, whereas DG and HSW markedly enhance heat evolution; additionally, HSW facilitates Friedel’s salt formation and pore filling, thereby improving matrix densification and strength development. Overall, this work provides a low-cost backfill solution and a transferable multi-objective optimization methodology for synergetic valorization of industrial solid wastes from multiple sources and HSW.

## Introduction

**Fig. 1:**
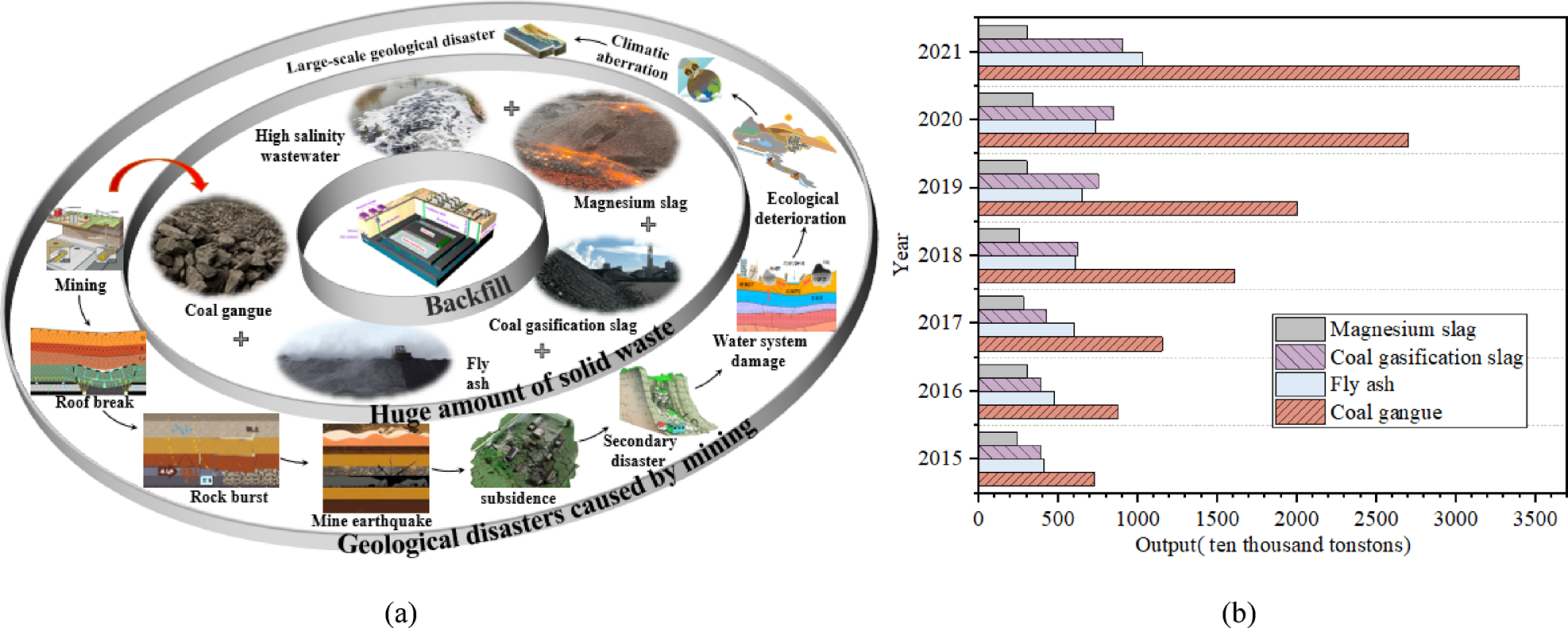
Regional advantages of backfill mining in Yulin. (a). (b) Major sources and quantities of solid waste generated in Yulin.

Coal continues to dominate the global energy mix and accounted for approximately 55.3% of total energy consumption of China in 2023^1–3^. While coal mining is pivotal in ensuring national energy security, it also brings about severe environmental challenges. The surface accumulation of coal gangue poses risks of soil contamination, and large-scale mining activities often trigger surface subsidence and damage to groundwater systems^4–7^. To promote the clean, efficient utilization of coal, the coal-chemical industry has developed rapidly in recent years. However, this growth has produced huge amount of industrial solid waste and wastewater, creating new environmental burdens^8^. In China, the annual production of coal gasification slag (CGS) exceeds 50 million tons, with approximately 3 million tons of magnesium slag produced in the Yulin region alone^9–11^. These waste streams are also accompanied by considerable volumes of high-salinity wastewater (HSW) discharge^12^, as illustrated in Fig. 1(a). Against this backdrop, backfill mining has become a pivotal technique for developing the green mining. By utilizing locally available industrial solid wastes to produce low-cost and eco-friendly total solid waste (TSW) backfill materials, this approach not only mitigates geological hazards but enables utilization of massive solid waste resources at the large scale as well, offering a viable pathway for sustainable development in major energy bases, as illustrated in Fig. 1(b)^13,14^.

Cementitious binders serve as the cornerstone of backfill technology. However, traditional cement-based binders face problems including the high energy consumption, elevated carbon emissions, and substantial production costs^14^. Consequently, the development of novel binder systems derived from region-specific industrial solid wastes has become a major focus of recent research efforts^15^. A growing body of research has paid attention to development of backfill materials based on solid waste. Li Jijian et al. utilized fly ash, steel slag, and desulfurized gypsum to prepare cementitious backfill, reporting that increased dosages of gypsum and fly ash enhanced compressive strength, with ettringite identified as the dominant hydration product^13,15^. Wentao Xia et al.^16^. formulated a multi-source coal-based backfill material by combining coal gangue, gasification slag, and fly ash, and systematically investigated how individual components influence strength development and microstructural evolution. Ruan Shishan et al.^17^. developed a backfill based on modified magnesium slag (MMS) by incorporating aeolian sand, achieving favorable mechanical and environmental performance, with peak strength reaching 17.14 MPa at the 300th day. Yujing Wang et al.^18^. explored how the concentration of sulfate ions in high-salinity mine water affects hydration mechanisms and mechanical behaviors of the gangue-based backfill material. However, in the practical engineering application, backfill materials generally need to simultaneously satisfy multiple performance criteria, particularly the strength and workability. As such, optimizing the mix proportions has become a critical challenge, and researchers have increasingly approached it as a multi-objective optimization (MOO) problem. For instance, Xiaoxiang Zhang et al.^19^. analyzed effects of mix parameters such as water-to-binder ratio on the CO₂ sequestration and mechanical performance of carbonized steel slag bricks by employing the response surface methodology (RSM). By integrating RSM with a multi-objective particle swarm optimization algorithm, Zhang Lanfang et al.^20^. studied synergistic effects of hybrid fibers and steel slag upon mechanical and conductive properties of cementitious composites. Yong Li et al.^21^. combined RSM and the NSGA-III algorithm to optimize mix designs for hydraulic asphalt concrete, targeting both cost-efficiency and carbon reduction. However, existing studies on multi-objective optimization for backfill materials still exhibit notable limitations.Most studies rely on subjective weighting methods such as the Analytic Hierarchy Process in the optimal solution decision-making phase, which inevitably introduces human-induced biases.In particular, systematic research on multi-objective optimization and comprehensive decision-making for synergistic backfill systems incorporating multi-source industrial solid wastes remains relatively scarce.

Despite significant progress, existing studies often fall short in addressing the coordinated use of solid wastes from multiple sources and the balanced optimization of multiple performance indicators. To fill in this gap, a TSW-based backfill material was developed using MMS, coarse gasification slag, DG, and HSW. Compressive strength and slump were selected as core performance targets. Based on RSM and constraint-based modeling, objective functions were constructed and validated via ANOVA. A Pareto optimal set of mix designs was obtained using the NSGA-III algorithm, and further evaluated using the entropy-weighted TOPSIS method to identify optimal proportions for different workability intervals. Finally, hydration heat and microstructure analyses were conducted to elucidate the material’s hydration mechanism and strength development. The research provides a theoretical basis for low-cost valorization of industrial solid waste and offers a methodological reference for multi-objective mix design optimization.

## Materials and methods

### Materials and characterization

#### Aggregate

**Fig. 2:**
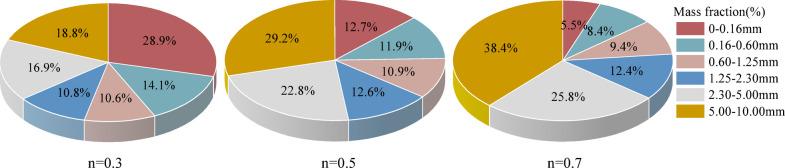
Effect of gradation index on particle-size distribution of coal gangue.

Aggregate was sourced from a coal mine in Yulin City, Shaanxi Province, China. Based on the Taylor gradation formula (Eq. 1), multiple gradation indices were selected. After being crushed, the gangue was screened into discrete particle-size ranges. According to the Talbot grading theory, the mass fractions of coal gangue within six particle-size intervals:0–0.16, 0.16–0.60, 0.60–1.25, 1.25–2.30, 2.30–5.00, and 5.00–10.00 mm—were calculated. Figure 2 shows gradation results. The maximum particle size is denoted as $\:{X}_{max}$, and the $\:M/{M}_{t}$ ratio, representing the ratio of cumulative mass of particles with diameters smaller than or identical to * x* to the total mass $\:{M}_{t}$, was determined according to the gradation theory^22^.

1 $$\frac{M}{{{M_{\mathrm{t}}}}}={\left[ {\frac{x}{{{x_{\hbox{max} }}}}} \right]^n}$$

The mass of particles with sizes falling within the interval [*x*_*1*_,*x*_*2*_]

2 $${M_1}=\left[ {{{\left( {\frac{{{x_2}}}{{{x_{\hbox{max} }}}}} \right)}^n} - {{\left( {\frac{{{x_1}}}{{{x_{\hbox{max} }}}}} \right)}^n}} \right]{M_{\mathrm{t}}}$$

In the equation, *n* represents the Taylor gradation index, which was set to 0.3, 0.5, and 0.7 in this study.

Coal gangue primarily contains kaolinite and quartz, characteristic of sandstone and argillaceous rock types, as illustrated in Fig. 4(a). Secondary mineral phases include illite and montmorillonite, while calcareous minerals such as limestone and calcite are present in relatively minor amounts^23^. Mineralogical analysis indicates that the gangue from the Mahuangliang coal mine is mainly composed of sandstone and kaolinite, with low contents of illite and montmorillonite. The material exhibits high hardness, low expansibility, and strong resistance to disintegration in water.

#### Cementitious binders

**Fig. 3:**
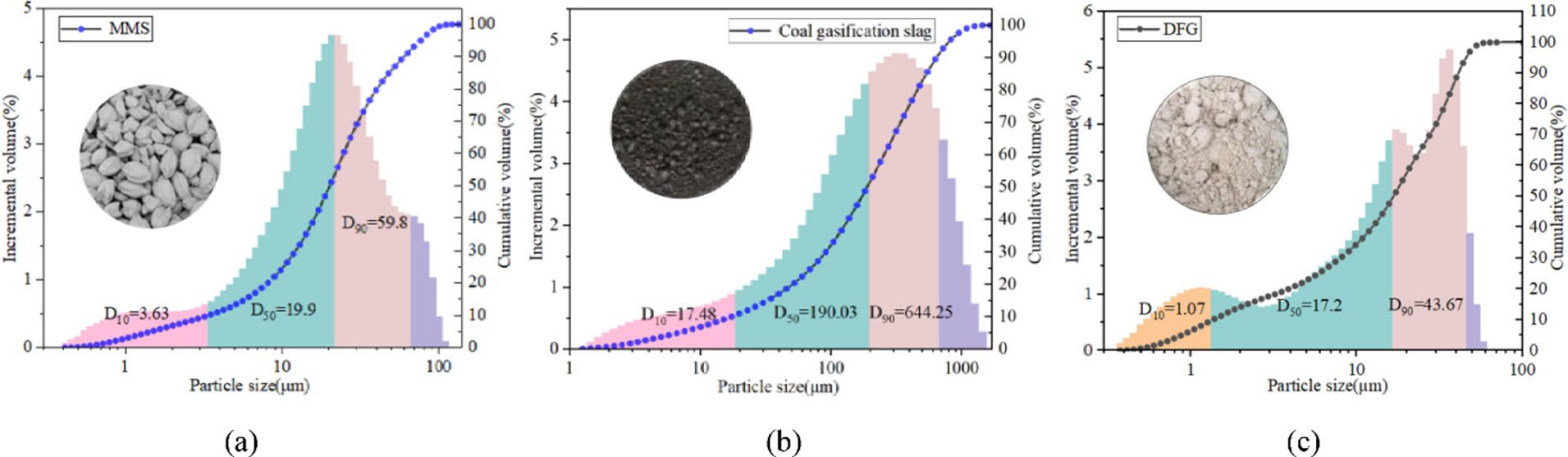
Particle-size distribution curves of the raw materials. (**a**) MMS, (**b**) CGS, (**c**) DG.

The cementitious binder utilized in this study was formulated by dry-blending MMS and CGS at a mass ratio of 1:9. MMS and CGS were separately sourced from Dongfeng Magnesium Industry Co., Ltd. and China National Coal Chemical Corporation, both located in Yulin, China. The magnesium slag was modified through Pidgeon process, in which chemical stabilizers were introduced to alter the crystal structure and polymorph of dicalcium silicate (C₂S), with the dual aim of enhancing reactivity and mitigating the powdering tendency commonly associated with untreated slag^24,25^. Following modification, the MMS exhibited a granular morphology with particle sizes ranging from 3 to 4 cm. Subsequent grinding yielded a fine powder with a particle-size distribution characterized by D₁₀ = 6.63 μm, D₅₀ = 19.9 μm, and D₉₀ = 59.8 μm (Fig. 3(a)). For MMS, the principal mineralogical components were SiO₂ and CaO, and its alkalinity coefficient was calculated to be 2.38, indicating that the material possesses high latent hydraulic reactivity and is suitable for use as an alkaline industrial by-product in binder systems.

**Fig. 4:**
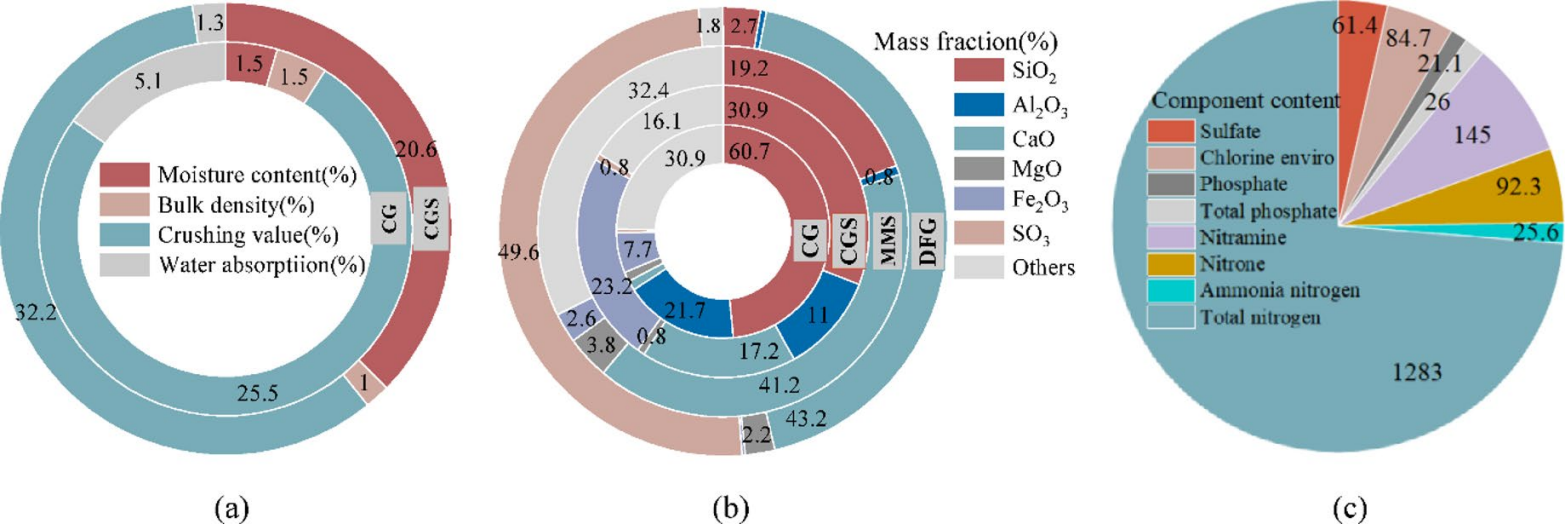
Fundamental physicochemical properties of the raw materials. (**a**) Physical characteristics, (**b**) Chemical composition, (**c**) Composition of HSW.

The as-received CGS exhibited a shiny black, vitreous appearance and a relatively high moisture content, necessitating oven drying prior to milling. As illustrated in Fig. 3(b), more than 96.6% of the CGS particles were coarser than 4 mesh. The chemical composition across particle-size fractions is presented in Fig. 4(b). The material was rich in aluminosilicate and calcium-bearing phases, primarily Al₂O₃, CaO, Fe₂O₃, and SiO₂, which together accounted for over 50 wt%. The carbon content exhibited an inverse correlation with particle size. According to the Chinese national standard *Fly Ash Used for Cement and Concrete* (GB/T 1596–2017), the acceptable threshold for loss on ignition (LOI) is 8%. Analytical results showed that CGS particles passing through a 24-mesh sieve had a carbon content of 3.53 wt%, and this fraction constituted 51.84% of the total sample mass. This size fraction was therefore selected and retained for use in subsequent mixture formulations^26^.

#### Activator and high-salinity water

Desulfurization gypsum (DG) (Guohua Power Plant in Yulin, China) was used as the chemical activator. The material exhibited a light yellow appearance and a moisture content of approximately 12.34%. It was incorporated into the binder system at a dosage of 3% by mass. The particle-size distribution of DG predominantly ranged between 5 μm and 50 μm, with measured D₁₀, D₅₀, and D₉₀ values separately of 2.74 μm, 15.03 μm, and 49.43 μm, as depicted in Fig. 3(c). The primary chemical constituents of DG were SO₃ and CaO, along with minor proportions of SiO₂, Al₂O₃, and MgO.

The high-salinity water (HSW) used in the mixture primarily consisted of chloride salts, as illustrated in Fig. 4(c). This brine-type wastewater was introduced not only as a mixing medium but also as a reactive component influencing the hydration and development of microstructures in this binder system.

### The framework for performance control

#### RSM

RSM is an experimental design approach that balances efficiency and accuracy in model development. RSM-based experiments were carried out adopting Box–Behnken Design (BBD) implemented in Design-Expert software^27,28^. Three critical factors influencing compressive strength and workability—namely, slurry mass concentration, aggregate-to-cement ratio, and aggregate grading index—were selected as independent variables (separately expressed as A, B, and C). These variables were assigned three levels: mass concentrations of 72, 76, and 80 wt%; aggregate-to-cement ratios of 1:1, 3:2, and 1:2; and gradation indices of 0.3, 0.5, and 0.7.

**Fig. 5:**
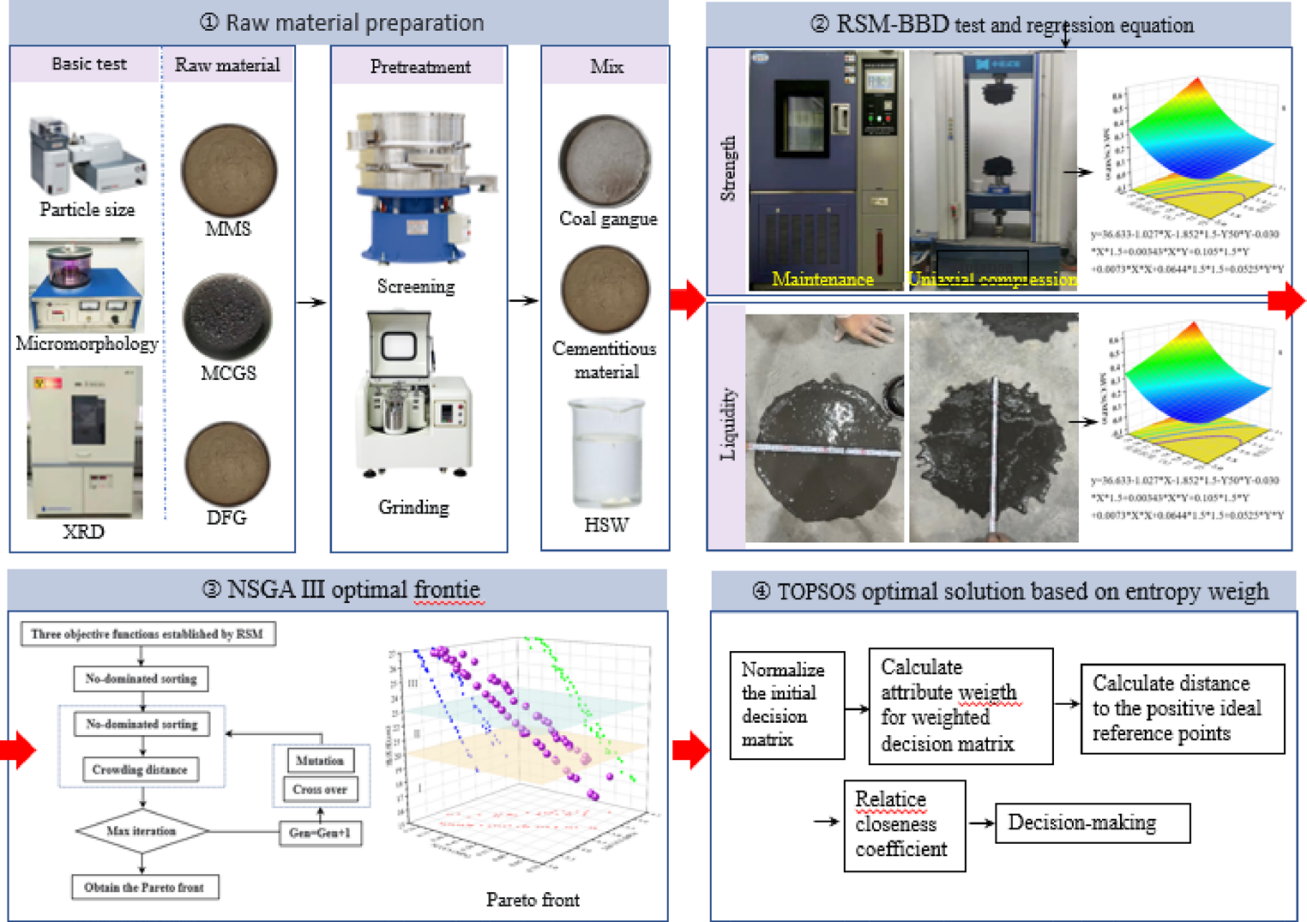
Experimental procedure and analysis framework.

The dependent variables (responses) included the 3-, 14-, and 28-day uniaxial compressive strengths (UCSs) (denoted as *Y₁*, *Y₂*, and *Y₃*, respectively), as well as the initial slump of the fresh backfill slurry (*Y*₄). The interactive and individual effects of independent variables upon the performance of the TSW backfill material were investigated through 17 experimental runs (Table 1). Figure 5 shows the experimental procedure.

Prior to testing, all raw materials were characterized in terms of particle-size distribution and physicochemical properties. Slurry mixtures were prepared according to the RSM design matrix under standardized mixing and curing conditions. Slump tests were performed on fresh mixtures, and samples were cast for subsequent uniaxial compressive strength testing at specified curing ages. Finally, the optimal mix proportions were determined using a combination of the entropy-weighted TOPSIS method and the NSGA-III MOO algorithm.

**Table 1:** RSM–BBD factor levels.

Factor	Horizontal coding		
	−1	0	1
Mass concentration (x_1_)	72	76	80
Aggregate-to-cement ratio (x_2_)	1	1.5	2
Aggregate grading index (x_3_)	0.3	0.5	0.7

#### NSGA-Ⅲ global optimization

**Fig. 6:**
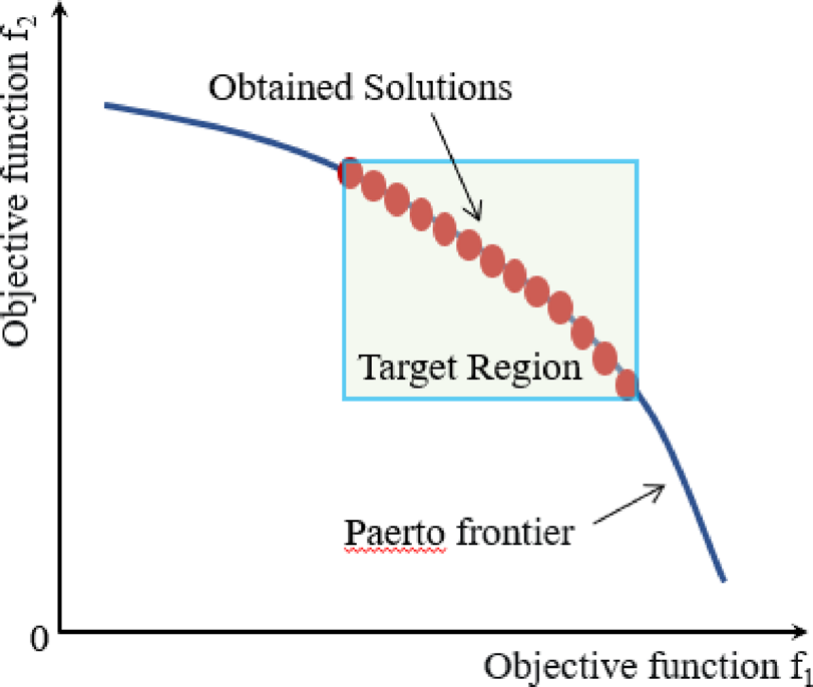
Schematic for results of the MOO algorithm.

NSGA-III is a reference-point–based evolutionary algorithm derived from non-dominated sorting applied to MOO (many-objective optimization). It represents the preference region by the ranges of individual objectives and conducts selection based on the distance to a set of fixed reference points, thereby yielding Pareto-optimum solutions with good convergence and a near-uniform distribution within the defined region (Fig. 6)^29,30^. Notably, when the number of objectives is ≥ 4, NSGA-III generally exhibits advantages in maintaining solution diversity and achieving a more even spread along the Pareto front.

Slurry workability and backfill strength at different curing ages are among the most critical indicators for evaluating backfilling performance. Accordingly, adjusting the mix proportions to simultaneously satisfy the required backfilling performance while minimizing cost constitutes the primary objective of mine backfill mix design. However, workability and strength are typically conflicting responses: in general, a higher solid mass concentration and a higher aggregate-to-cement (or sand-to-cement) ratio enhance the compressive strength of the backfill, yet deteriorate slurry flowability. In addition, the aggregate grading index often exhibits an optimum for both strength and flowability, which is primarily governed by the spatial distribution and packing state of aggregates and binders within the slurry.

In this study, the strength is quantified by targeting the maximization of UCS at the 3rd, 14th, and 28th day, serving as key indicators of early-age trafficability/working-platform capacity and later-stage load-bearing performance. Workability is constrained by the target slump ranges of 15–20 cm (pumping) and 23–27 cm (self-flowing). Based on these considerations, an MOO model is formulated below:

#### TOPSIS model based on entropy weight

The entropy-weighted technique for order preference by similarity to ideal solution (TOPSIS) approach is essentially a distance-to-ideal-solution method. Its core is to derive objective weights for each evaluation indicator based on information entropy, and then incorporate these weights into the TOPSIS procedure by weighting the normalized indicator matrix. In this way, the method can make full use of the original data and more effectively capture the performance gaps among alternative schemes. The specific steps are summarized as follows^31,32^. In contrast to conventional subjective weighting methods, the entropy-weighted TOPSIS model objectively derives indicator weights from raw data, eliminating subjective preference-induced biases and ensuring the objectivity and scientificity of decision-making. It enables quantitative ranking of alternatives under mutually constrained multi-performance indicators, well-suited for multi-objective optimization of backfill material mixture proportions.

1. The evaluation indicators are normalized according to Eq. (3).

3 $$x_{{ij}}^{\prime }=\frac{{{x_{ij}}{\mathrm{-}}x_{j}^{{\hbox{min} }}}}{{x_{j}^{{\hbox{max} }}{\mathrm{-}}x_{j}^{{\hbox{min} }}}},x_{{ij}}^{\prime }=\frac{{x_{j}^{{\hbox{max} }}{\mathrm{-}}{x_{ij}}}}{{x_{j}^{{\hbox{max} }}{\mathrm{-}}x_{j}^{{\hbox{min} }}}}$$

(2) A numerical translation (shift) is performed to eliminate potential negative values or zeros.

4 $$x_{{ij}}^{{''}}{\mathrm{=}}H+x_{{ij}}^{\prime }$$

(3) Dimensionless processing is conducted using the proportion (ratio) method.

5 $${y_{ij}}=\frac{{x_{{ij}}^{{''}}}}{{\sum\limits_{{i=1}}^{n} {x_{{ij}}^{{''}}} }}$$

(4) The entropy value of the (j)^th^ indicator is calculated.

6 $${e_j}= - \frac{1}{{\ln n}}\sum\limits_{{i=1}}^{n} {{y_{ij}}} \ln {y_{ij}}$$

(5) The divergence (variation) coefficient of the (j)^th^ indicator is given by:

7 $${g_j}=1 - {e_j}$$

(6) The weight of the (j)^th^ indicator is determined and a weighted normalized decision matrix constructed.

8 $${\omega _j}=\frac{{{g_j}}}{{\sum\limits_{{j=1}}^{p} {{g_j}} }},{c_{ij}}={\omega _j}{b_{ij}}$$

(7) The positive-ideal and negative-ideal solutions (C^*^ and *c*^*o*^) are determined:

9 $$c_{j}^{*}=\mathop {\hbox{max} {c_{ij}}}\limits_{i} j=1,2,\; \cdots n,c_{j}^{0}=\mathop {\hbox{min} {c_{ij}}}\limits_{i} j=1,2,\; \cdots n.$$

(8) Euclidean distances of each alternative from positive-ideal and negative-ideal solutions are computed, with the comprehensive evaluation index *C*_*i*_ obtained as follows:

10 $${d_i}^{*}={[\sum\limits_{{j=1}}^{n} {({c_{ij}} - \mathop {\hbox{max} }\limits_{i} } \{ {c_{ij}}\} )^2}{]^{1/2}},{d_i}^{0}={[\sum\limits_{{j=1}}^{n} {({c_{ij}} - \mathop {\hbox{min} }\limits_{i} } \{ {c_{ij}}\} )^2}{]^{1/2}}$$

(9) The comprehensive evaluation index *C*_*i*_ is then derived.

11 $${C_i}=\frac{{{d_i}^{0}}}{{{d_i}^{0}+{d_i}^{*}}}$$

### Test content and method

#### Sample preparation

All constituent materials for each mix were weighed using an electronic balance in accordance with the prescribed proportions. The materials were then charged into a mixer and blended until forming a homogeneous slurry, followed by casting the slurry into standard cube molds (70.1 mm × 70.1 mm × 70.1 mm). Prior to casting, a thin layer of silicone oil/agent immiscible with the slurry was smeared onto the inner surface of these molds to facilitate demolding^33^. The specimens were labeled and cured in a standard curing chamber at a temperature of (20 ± 1) °C and relative humidity of (95 ± 1)% for the designated curing ages. The specimen geometry, mixing procedure, and curing conditions conformed to the relevant Chinese standards JG 237–2008, JG/T 3033 − 1996, and GB/T 50,081 − 2002, respectively^34^.

#### Workability (Slump)

The slump test, originally developed in concrete engineering, has been extensively applied to assessment of flow state and frictional resistance of backfill slurry. Its underlying mechanical principle is that the slurry deforms under self-weight and ceases flowing when the internal resistance balances the driving force, with the final deformation taken as the slump value. In this study, a standard conical slump mold was employed, of which the top diameter, bottom diameter, and height were 100 mm, 200 mm, and 300 mm, respectively. The test procedure followed relevant specifications in *Test Methods for Properties of Fresh Ordinary Concrete Mixtures* (GB/T 50080 − 2016). The main apparatus included a slump cone, charging funnel, dedicated ruler, and tamping rod^35^.

#### UCS

The UCS of TSW-CPB specimens was tested following GB/T 17,671 − 1999 after reaching the target curing ages. An MTSC43.504 electronic universal tester was used, loading in the displacement-controlled mode at rate of 1 mm/min^36^. Stress–strain data were recorded for each specimen. To reduce experimental uncertainty, three replicates were tested for each mix at each curing age, and the average UCS was calculated for subsequent analysis. The “Three-Underground” mining specification stipulates that grouted backfills in goafs beneath Class A and B buildings with high protection requirements should have a strength no less than 2 MPa.

#### Hydration reaction

The eight-channel isothermal microcalorimeter (TAM Air) was adopted to characterize the hydration heat evolution of the backfill materials at 20 °C. This instrument enables continuous, high-precision recording of the heat-flow rate during hydration. The test procedure was as follows: the total heat capacity of the tested sample was calculated based on the mix design, and distilled water with an equivalent heat capacity was used as the reference medium. The reference medium was weighed into a glass ampoule and placed in the reference channel so that the test channel remained at approximately 20 °C. After being weighed utilizing an analytical balance (precision 0.0001 g), the dry constituents were mixed for 3 min and immediately transferred into the test channel. After recording heat-flow signals real-timely, the cumulative heat release was acquired by integration^37^.

#### SEM and XRD analyses

For scanning electron microscopy (SEM), a small fragment was collected from the core of fractured specimens following UCS tests, prepared following standard procedures, and used to examine the microstructural features of specimens with different mix proportions. For the purpose of identifying crystalline phases of raw materials and specimens with different mix designs and curing ages, X-ray diffraction (XRD) was performed. Sample preparation for XRD was generally similar to that for SEM, except that gold coating was not required. An X-ray diffractometer (Rigaku D/Max-3B) was adopted for Phase analysis. For the XRD parameters, Cu target radiation was used, and the scanning rate and range were set to be 8°/min and 5°–80° (2θ), respectively^36,37^.

## Results and discussion

### Development and validation of response surface models

#### Model establishment

Based on the least-squares principle, regression analysis was performed for experimental results **in** Table 1 **to** develop models for predicting the 3-, 14-, and 28-day UCSs of TSW-CPB, as well as the slump of the fresh slurry. The F-test was conducted to assess statistical significance of various models. At a 95% confidence level, a P-value less than 0.05 suggests statistical significance of a model; a smaller P-value indicates a higher level of significance. Following this criterion, non-significant terms were eliminated via stepwise regression to improve the predictive accuracy of the models. The least-squares method was used to fit responses at the designed experimental points (Table 1) to the second-order response surface model^38^.

12 $$\left\{ {\begin{array}{*{20}{c}} {{Y_1}=0.1698+0.1566{X_1} - 0.1173{X_2} - 0.0134{X_3} - 0.0610{X_1}{X_2}+0.0027{X_1}{X_3} - 0.0055{X_2}{X_3}+0.1045X_{1}^{2}+0.0287X_{2}^{2}+0.0145X_{3}^{2}} \\ {{Y_2}=0.7348+0.5299{X_1} - 0.3694{X_2} - 0.1060{X_3} - 0.2637{X_1}{X_2} - 0.0830{X_1}{X_3} - 0.0170{X_2}{X_3}+0.1360X_{1}^{2}+0.0845X_{2}^{2}+0.0597X_{3}^{2}} \\ {{Y_3}=2.54+1.47{X_1} - 0.7550{X_2}+0.0819{X_3}+0.1752{X_1}{X_2}+0.2210{X_1}{X_3} - 0.0488{X_2}{X_3}+0.9720X_{1}^{2} - 0.2637X_{2}^{2}} \\ {{Y_4}=28.99 - 4.85{X_1}+1.33{X_2}+0.5560{X_3}+01.08{X_1}{X_2} - 0.8770{X_1}{X_3} - 3.62X_{1}^{2} - 0.6242X_{2}^{2}+1.23X_{3}^{2}} \end{array}} \right.$$

In the equation, *X*_*1*_ denotes the mass concentration, *X*_*2*_ is the aggregate-to-cement ratio, and *X*_*3*_ is the aggregate grading index; *Y*_*1*_, *Y*_*2*_, and *Y*_*3*_ represent the 3-, 14-, and 28-day UCSs, respectively, and *Y*_*4*_ denotes the slump value.

**Table 2:** Experimental results.

No.	Factor			Test result			
	Mass concentration (%)	Aggregate-to-cement ratio	Grading index	3-d UCS (MPa)	14-d UCS (MPa)	28-d UCS (MPa)	Slump (cm)
1	80	1.5	0.7	0.19	0.92	2.476	25.3
2	72	1.5	0.7	0.232	1.428	2.602	26.3
3	76	2	0.7	0.139	0.782	1.965	27.4
4	72	1	0.5	0.212	1.185	2.639	25.5
5	72	2	0.5	0.329	2.201	3.23	22.8
6	80	2	0.5	0.108	1.013	1.557	27
7	76	1	0.3	0.652	3.182	5.02	16.5
8	76	1.5	0.5	0.252	1.776	4.21	21.7
9	80	1	0.5	0.444	2.859	5.533	20.3
10	76	1.5	0.5	0.173	1.101	2.752	25.1
11	76	1.5	0.5	0.128	0.794	2.509	25.2
12	78	1.5	0.3	0.288	1.86	3.214	24.2
13	76	1.5	0.5	0.11	0.834	1.964	27.1
14	76	2	0.3	0.462	2.482	4.65	18.6
15	72	1.5	0.3	0.146	1.089	2.253	25.1
16	76	1	0.7	0.076	0.6	1.091	27.8
17	76	1.5	0.5	0.127	0.8	1.468	25.6

#### Test of model

To assess the accuracy and effectiveness of these models, each regression model was subjected to analysis of variance (ANOVA) using the F-test, as given in Eq. (13).

13 $$F=\frac{{{M_r}}}{{{M_e}}}=\frac{{{\raise0.7ex\hbox{${{S_{\mathrm{t}}}}$} \!\mathord{\left/ {\vphantom {{{S_{\mathrm{t}}}} v}}\right.\kern-0pt}\!\lower0.7ex\hbox{$v$}}}}{{{\raise0.7ex\hbox{${{S_e}}$} \!\mathord{\left/ {\vphantom {{{S_e}} {(n - v - 1)}}}\right.\kern-0pt}\!\lower0.7ex\hbox{${(n - v - 1)}$}}}}$$

In the equation, *M*_*r*_ is the mean square due to regression; *M*_*e*_ is the mean square error; *S*_*r*_ and *S*_*e*_ separately represent the regression sum and residual sum of squares; *n* is the number of experimental runs; *v* is the quantity of variables terms in the response model.

According to statistical criteria, a model is considered valid when calculated *F*-values exceed *F*_*0*_, that is, the critical value. The *P*-value represents the probability of obtaining the observed effect for each model term and is used to evaluate significance (highly significant for *P* < 0.01; significant for *0.01 ≤ P ≤ 0.05*; insignificant for *P* > 0.05). It can be seen from Tables 2, 3 that all four models show high significance (*P* < 0.0001), while the lack-of-fit is not significant (*P* > 0.05). The coefficients of determination *R*^*2*^ range from 0.9478 to 0.9740, indicating high reliability and good goodness-of-fit with solid statistical significance. In addition, the “Adeq Precision” values are much higher than 4, and the “Predicted *R*^*2*^” and “Adjusted *R*^*2*^” have a difference lower than 0.2, suggesting the close agreement of response surface regression models with experimental data and applicability of these models to further optimization and analysis^39,40^.

**Fig. 7:**
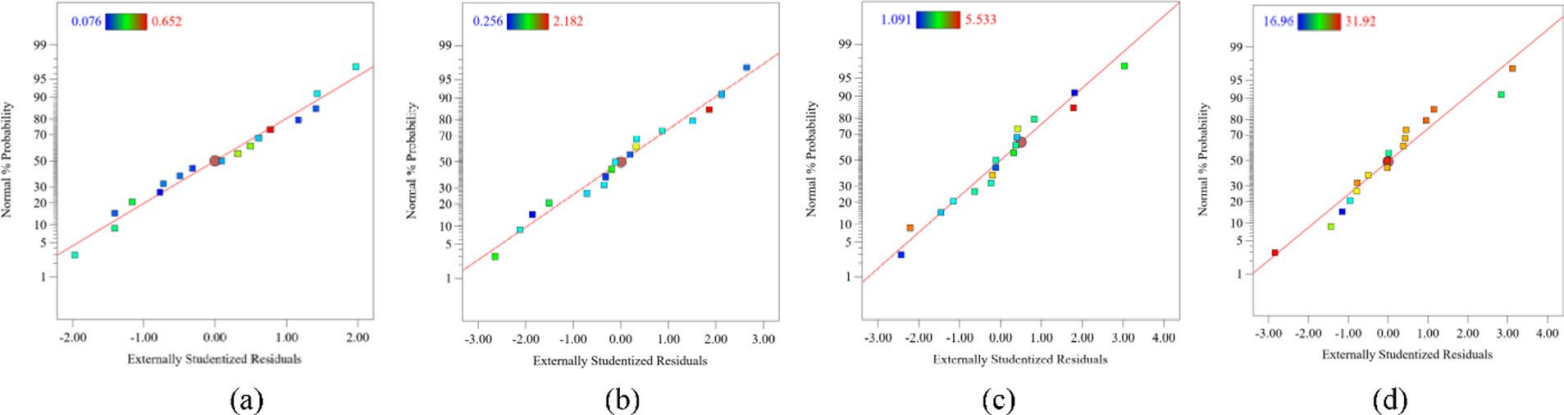
Residual analysis diagram: normal Probability Plot. (**a**) Y1, (**b**) Y2, (**c**) Y3, (**d**) Y4.

In the scatter diagram for predicted versus actual values (Fig. 7), the data points for each target variable (strength at different ages, slump) are closely distributed around the reference diagonal, showing a clear linear trend. This indicates that model predictions are highly aligned with actual observations, visually confirming the model’s excellent overall fitting accuracy and predictive capability for experimental results.

**Fig. 8:**
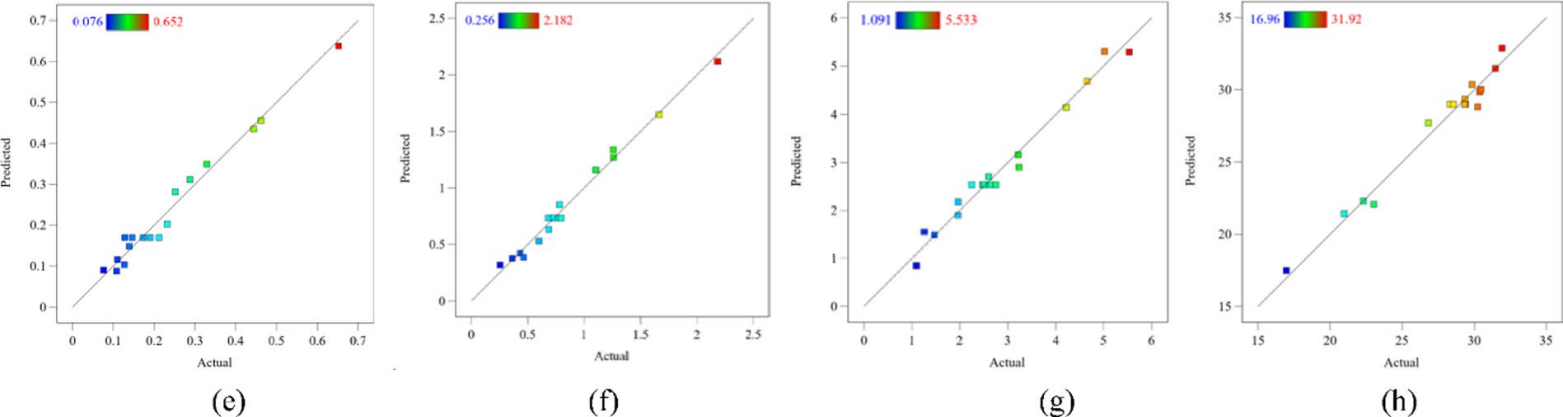
Residual analysis diagram: distribution of predicted and actual values. (**e**) Y1, (**f**) Y2, (**g**) Y3, (**h**) Y4.

In the plot for residuals versus predicted values (Fig. 8), the residuals for each target variable are randomly and uniformly distributed above and below the zero line, with no detectable systematic trend or heteroscedasticity. This result aligns with the classical assumptions of linear regression models, demonstrating that the selected model adequately explains the systematic variation in the data, with residuals containing only random noise, thereby confirming the statistical robustness and reliability of the model.

**Table 3:** ANOVA of the regression models.

Source	Sum of Squares				F-value				*P*-value			
	Y_1_	Y_2_	Y_3_	Y_4_	Y_1_	Y_2_	Y_3_	Y_4_	Y_1_	Y_2_	Y_3_	Y_4_
Model	0.3756	3.87	26.50	274.81	33.26	67.66	43.60	39.60	< 0.0001	< 0.0001	< 0.0001	< 0.0001
*X*_*1*_	0.1963	2.25	17.38	188.45	156.40	353.48	228.77	217.22	< 0.0001	< 0.0001	< 0.0001	< 0.0001
*X*_*2*_	0.1100	1.09	4.56	14.14	87.65	171.77	60.03	16.30	< 0.0001	< 0.0001	< 0.0001	0.0037
*X*_*3*_	0.0014	0.0899	0.0536	2.47	1.14	14.15	0.7059	2.85	0.3210	0.0071	0.4250	0.1298
*X*_*1*_ *X*_*2*_	0.0149	0.2783	0.1229	4.65	11.86	43.79	1.62	5.36	0.0108	0.0003	0.2392	0.0493
*X*_*1*_ *X*_*3*_	0.0000	0.0276	0.1954		0.0241	4.34	2.57	3.55	0.8810	0.0758	0.1475	0.0964
*X*_*2*_ *X*_*3*_	0.0001	0.0012	0.0095	3.08	0.0964	0.1819	0.1251		0.7652	0.6825	0.7327	
*X2 1*	0.0160	0.0778	3.99	55.06	36.63	12.25	52.51	63.47	0.0005	0.0100	< 0.0001	< 0.0001
*X2 2*	0.0035	0.0300	0.2937	1.64	2.77	4.73	3.87	1.89	0.1401	0.0662	0.0849	0.2064
*X2 3*	0.0009	0.0150		6.34	0.7031	2.36		7.30	0.4295	0.1681		0.0270
Residual	0.0088	0.0445	0.6077	6.94								
Lack of Fit	0.0043	0.0363	0.4666	5.89	1.26	5.89	3.31	5.58	0.3997	0.0598	0.1368	0.0623
Pure Error	0.0045	0.0082	0.1412	1.05								
R^2^	0.9772	0.9886	0.9776	0.9754								
Adjusted R_2_	0.9478	0.9740	0.9552	0.9507								
Predicted R^2^	0.8039	0.8485	0.8203	0.7911								
Adeq Precision	20.2442	29.4169	22.2284	22.7214								

### The influence of factors in the RSM on intensity

#### Main-effects analysis

**Fig. 9:**
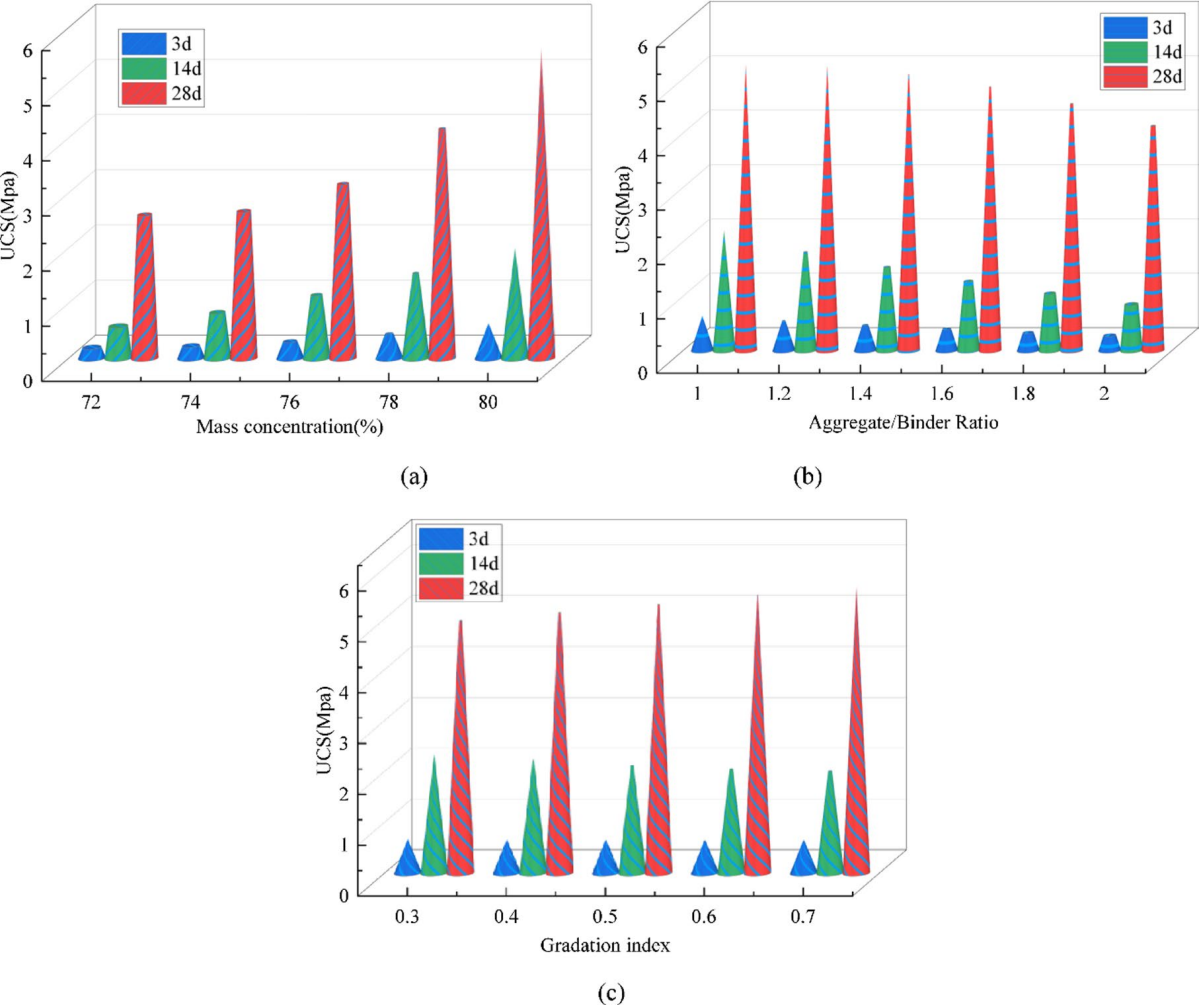
Main effects of individual factors on the UCS at different curing ages. (**a**) Mass concentration, (**b**) Aggregate-to-cement ratio, (**c**) 28-d UCS.

As indicated by Fig. 9 in conjunction with the ANOVA results of the RSM models in Table 2, individual factors exert significant effects on UCSs at various curing ages and slump of fresh slurry. For the UCS models at the 3rd, 14th, and 28th day, the P-values associated with the aggregate-to-cement ratio and the mass concentration are all < 0.0001, demonstrating their dominant influence on early-age strength development. In contrast, the P-values of the aggregate grading index are all > 0.005, suggesting a comparatively weaker contribution to early-age strength.

The main-effect trends of the mass concentration (*X*_*1*_), aggregate-to-cement ratio (*X*_*2*_), and aggregate grading index (*X*_*3*_) upon the UCS of TSW-CPB are presented in Fig. 9. When analyzing a single factor, the other two variables were held constant. Specifically, in the bar charts of Fig. 9(a–c), UCS variations were evaluated under fixed conditions of (*X*_*2*_ = 1.0) and (*X*_*3*_ = 0.5); (*X*_*1*_ = 80%) and (*X*_*3*_ = 0.5); and (*X*_*1*_ = 80%) and (*X*_*2*_ = 1.0), respectively. After being cured for 3, 14, and 28 day, the UCS is positively correlated with the mass concentration and negatively correlated with the aggregate-to-cement ratio, reaching its maximum 5.659 MPa when the mass concentration and aggregate-to-cement ratio are 80% and 1.0, respectively. When the mass concentration is 78%, the UCS of the backfill at different curing ages is 0.450 MPa, 1.549 MPa and 4.172 MPa, respectively. At 14 days and 28 days, the strength increased by 1.099 MPa (2.442 times) and 3.772 MPa (8.271 times), respectively, compared with that at 3 days.With the raw material system fixed, mass concentration becomes the primary determinant of UCS because it controls the solid-particle content and thus the packing density; a higher solids content promotes a denser overall structure^41,42^. Meanwhile, the binder fraction is the main source of strength in cemented backfill. With an increase in the aggregate-to-cement ratio, the binder content reduces, leading to reduced hydration product formation; consequently, microcracks and pores increase, resulting in strength degradation (Fig. 9b)^43,44^.

The influence of the aggregate grading index varies with curing age. At the 3rd day, UCS first decreases and then increases with increasing grading index (n). When the aggregate grading index n increases from 0.3 to 0.7, the corresponding UCS values are 0.668 MPa, 0.650 MPa, 0.638 MPa, 0.633 MPa and 0.636 MPa, which are reduced by 0.018 MPa, 0.030 MPa, 0.035 MPa and 0.032 MPa compared with the strength at *n* = 0.3.At the 14th day, UCS increases monotonically. At the same range of n from 0.3 to 0.7, the UCS values are 2.348 MPa, 2.219 MPa, 2.117 MPa, 2.048 MPa and 2.005 MPa, with respective reductions of 0.129 MPa, 0.231 MPa, 0.300 MPa and 0.343 MPa relative to the case of *n* = 0.3.Whereas at the 28th day UCS again exhibits a decrease–increase trend. As n rises from 0.3 to 0.7, the UCS values reach 4.962 MPa, 5.118 MPa, 5.283 MPa, 5.468 MPa and 5.661 MPa, which are higher than the strength at *n* = 0.3 by 0.156 MPa, 0.165 MPa, 0.506 MPa and 0.699 MPa in sequence.

**Fig. 10:**
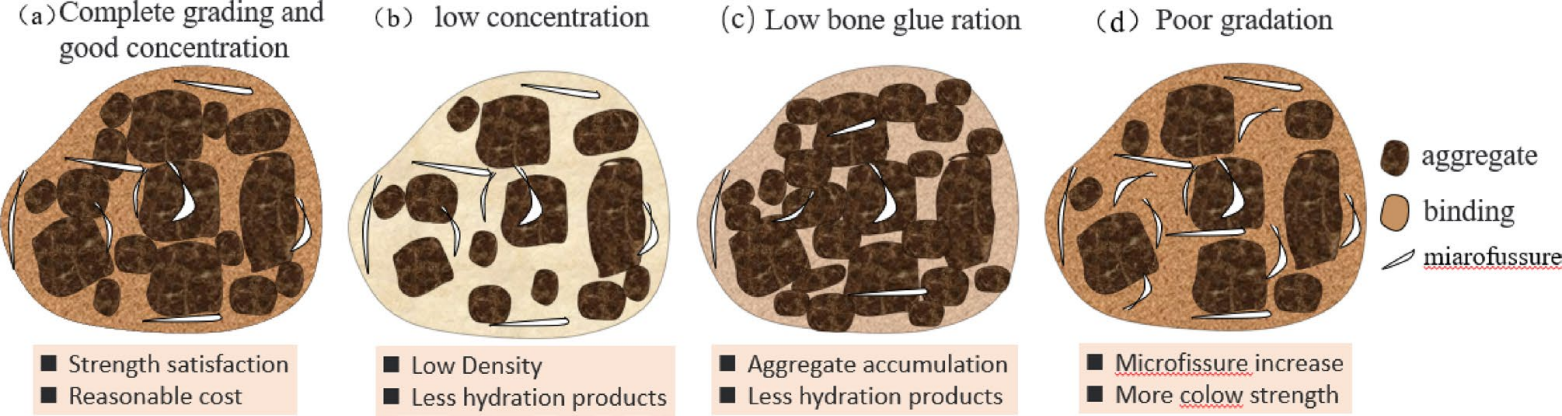
Effects of key factors on strength development.

This behavior is attributable to a time-dependent shift in the governing strength mechanism. At the 3rd and 14th day, strength is primarily controlled by particle packing and densification, where a lower grading index provides sufficient fines and a lower porosity, thereby favoring strength gain. As hydration proceeds, the 28-day strength becomes increasingly governed by skeletal load-bearing capacity. Under a higher grading index, coarse aggregates form a stiffer load-bearing framework, and once adequately cemented, their mechanical potential is fully mobilized (Fig. 9c), ultimately leading to strength “overtaking”^45,46^. These results indicate that the optimal grading should be selected according to the dominant mechanism associated with the target service age. A schematic illustration of the mechanistic roles of each factor is provided in Fig. 10.

**Fig. 11:**
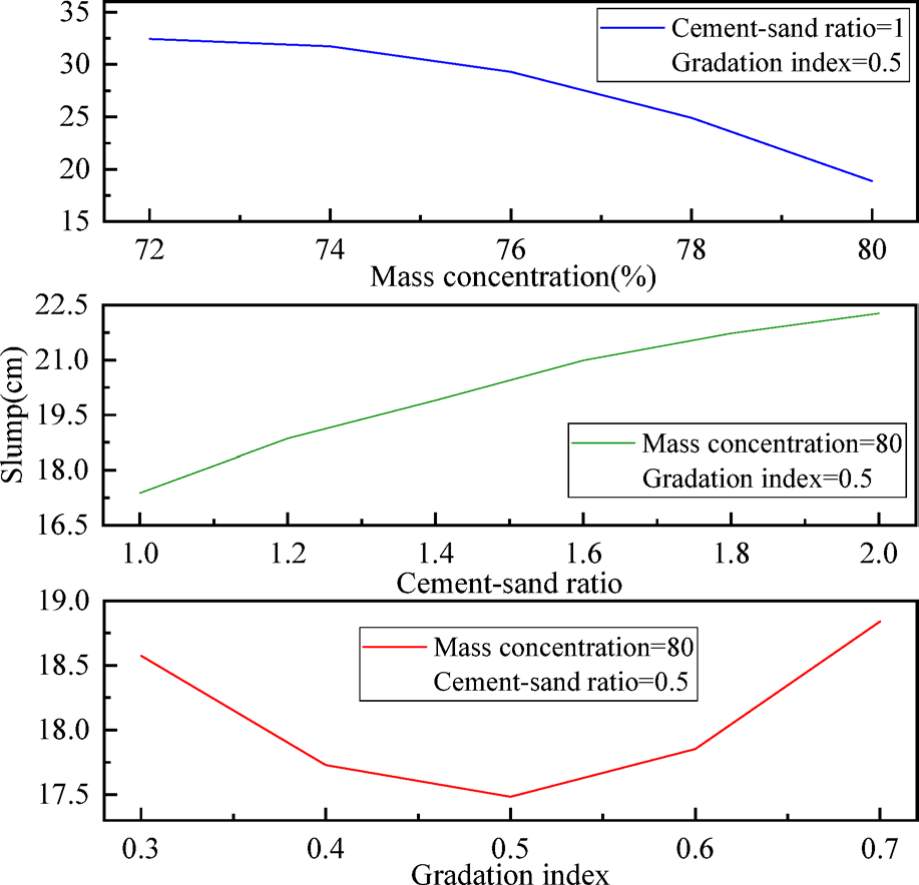
Main effects of individual factors on slump.

Figure 11 illustrates the single-factor effects on the slump of the fresh slurry. The slump is negatively correlated to the mass concentration and positively correlated with the aggregate-to-cement ratio. This is because the rising mass concentration reduces the thickness of the free-water film and strengthens interparticle van der Waals attraction, thereby markedly increasing plastic viscosity and yield stress of the fresh slurry. In comparison, increasing the aggregate-to-cement ratio mitigates direct interparticle friction by improving the paste-coating (wrapping) effect.

When the aggregate-to-cement ratio and mass concentration remain fixed, the slump decreases at first and then grows as the aggregate grading index increases, reaching a minimum at approximately (n \approx 0.5). At (*n* = 0.5), the aggregate system approaches its maximum packing density, resulting in the largest number of particle contact points and the strongest interlocking of the granular skeleton; consequently, internal friction opposing flow attains its peak, leading to the lowest slump. When the grading deviates from this near-optimal state ((*n* < 0.5) or (*n* > 0.5)), the porosity increases and the amount of excess paste rises sharply, such that lubrication becomes dominant and the slump improves substantially even with small changes in (n). Notably, the variation is asymmetric around the minimum: the rate of slump reduction prior to the minimum is smaller than the rate of slump increase after the minimum, i.e., a “slow-decrease–fast-increase” pattern.

Mechanistically, at (*n* = 0.5) the fines content is moderate and the packing structure is most compact, maximizing contact points and skeleton interlock and thus maximizing frictional resistance. When n decreases (higher fines content), although additional water demand of the aggregate may consume part of the free water, the excess fines fill intergranular voids and increase viscous resistance. Once a critical packing condition is exceeded, the rheology becomes increasingly governed by fines–fines interactions, and the effective resistance can drop abruptly, resulting in a rapid increase in slump. Conversely, when (n) increases (lower fines content), the relative proportion of coarse aggregate increases; however, due to the typically lower water absorption of coarse particles compared with fine particles, the deterioration of paste coating is less pronounced, and the packing void ratio changes more gradually, causing flow resistance to experience a net reduction, along with the increased slump correspondingly.

#### Interaction-effect analysis

**Fig. 12:**
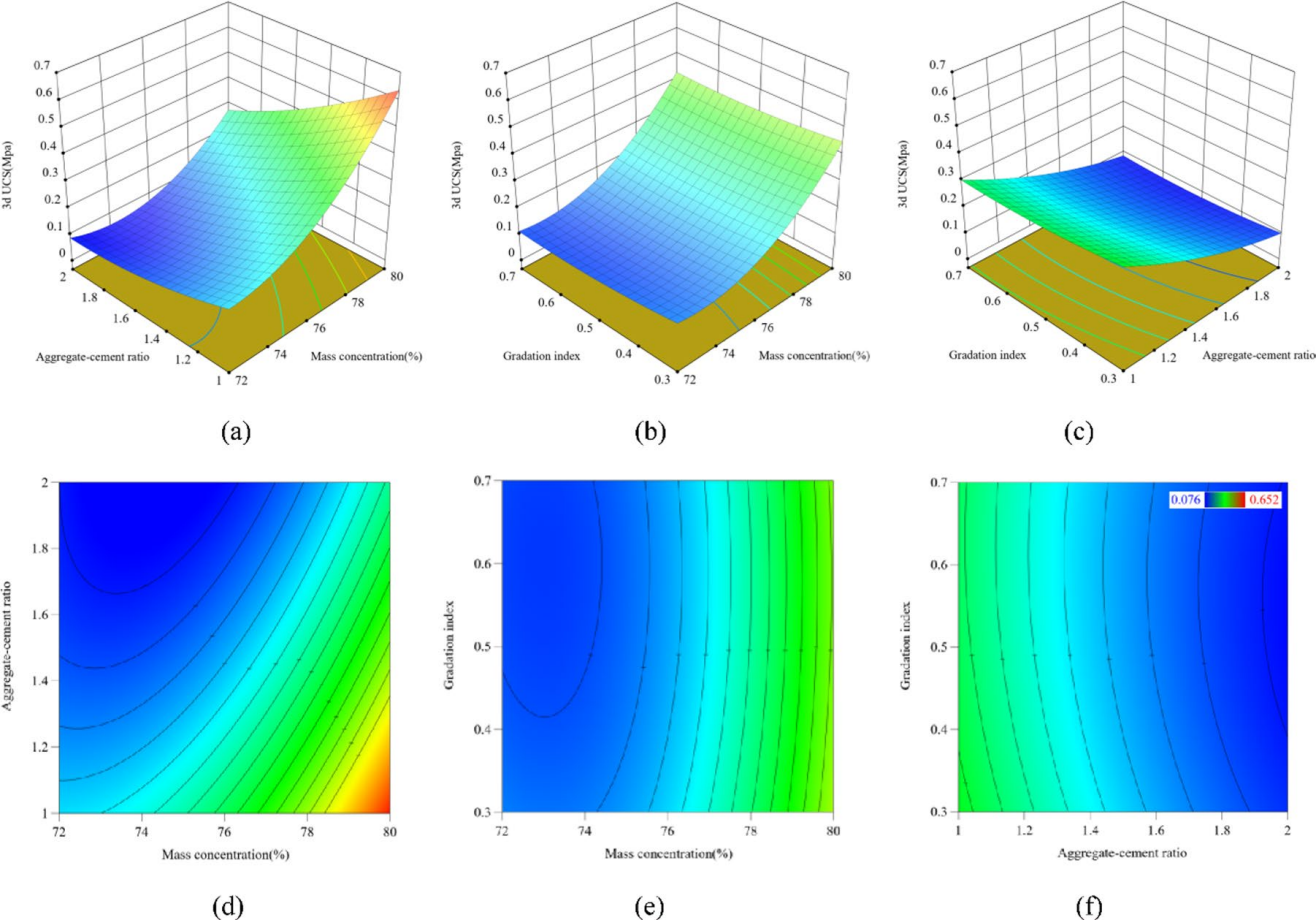
Response surface plots of 3-d UCS: (**a**) and (**d**) mass concentration and aggregate-to-cement ratio; (**b**) and (**e**) mass concentration and grading index; (**c**) and (**f**) aggregate-to-cement ratio and grading index.

**Fig. 13:**
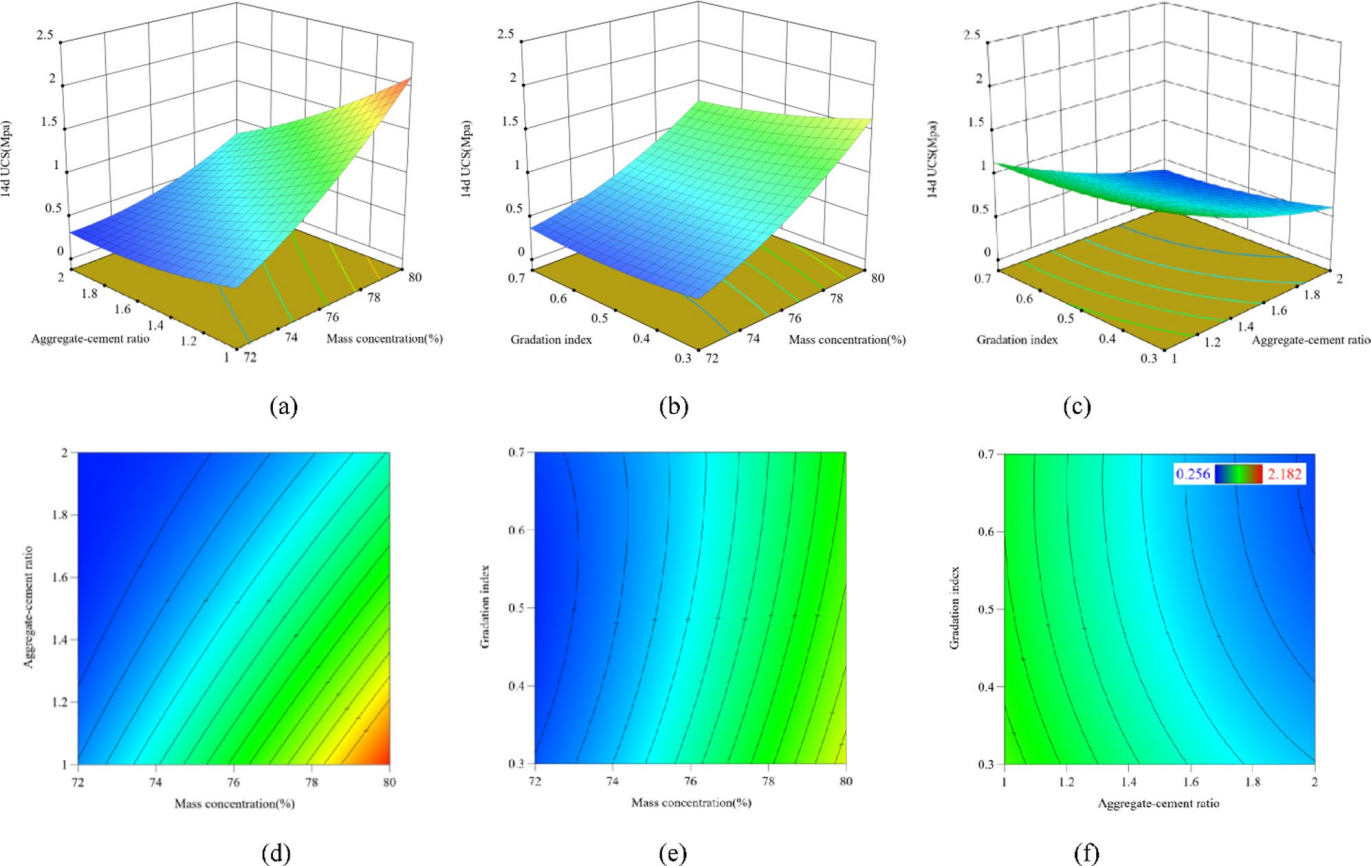
Response surface plots of 14-d UCS: (**a**) and (**d**) mass concentration and aggregate-to-cement ratio; (**b**) and (**e**) mass concentration and grading index; (**c**) and (**f**) aggregate-to-cement ratio and grading index.

**Fig. 14:**
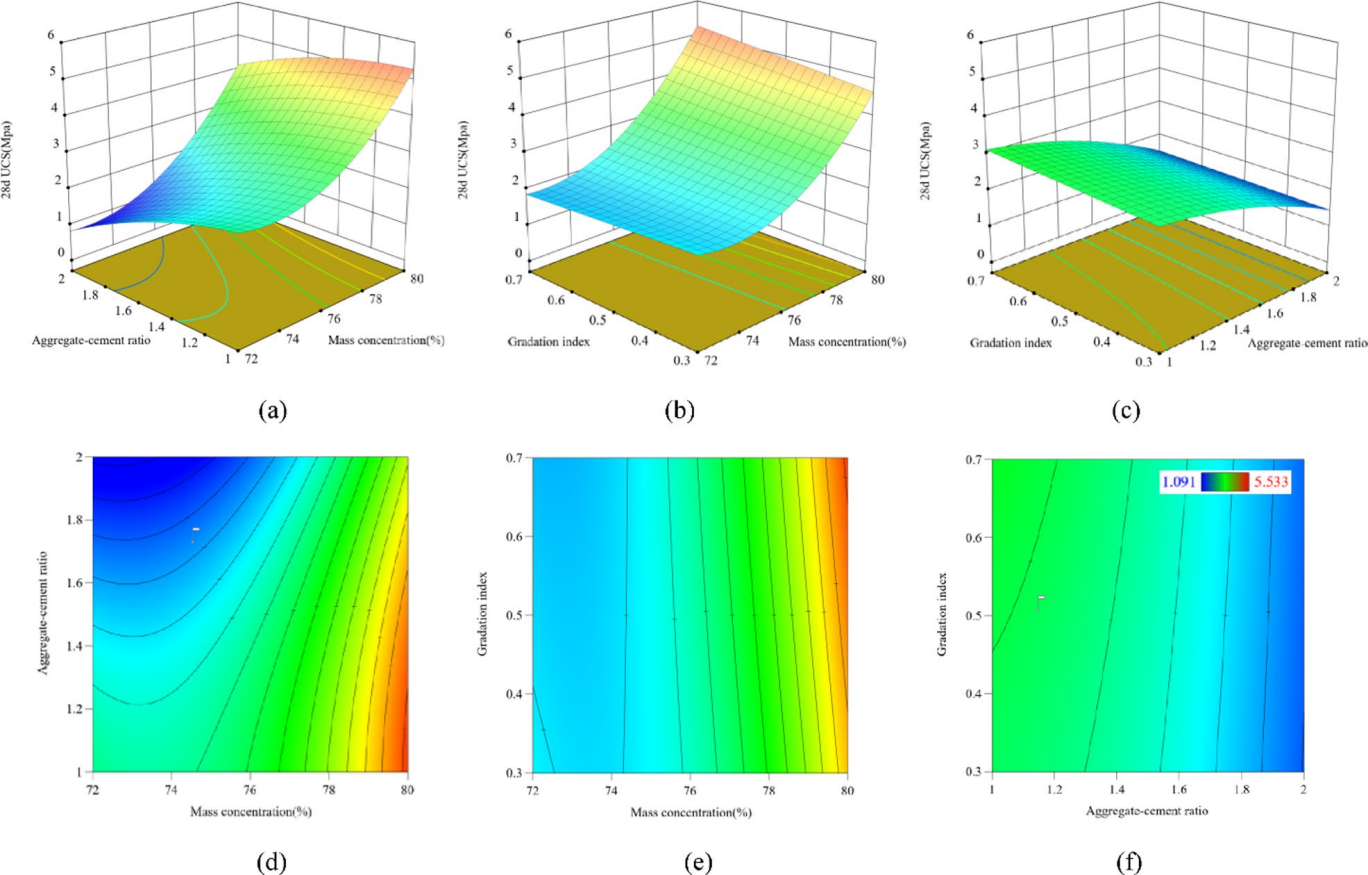
Response surface plots of 28-d UCS: (**a**) and (**d**) mass concentration and aggregate-to-cement ratio; (**b**) and (**e**) mass concentration and grading index; (**c**) and (**f**) aggregate-to-cement ratio and grading index.

**Fig. 15:**
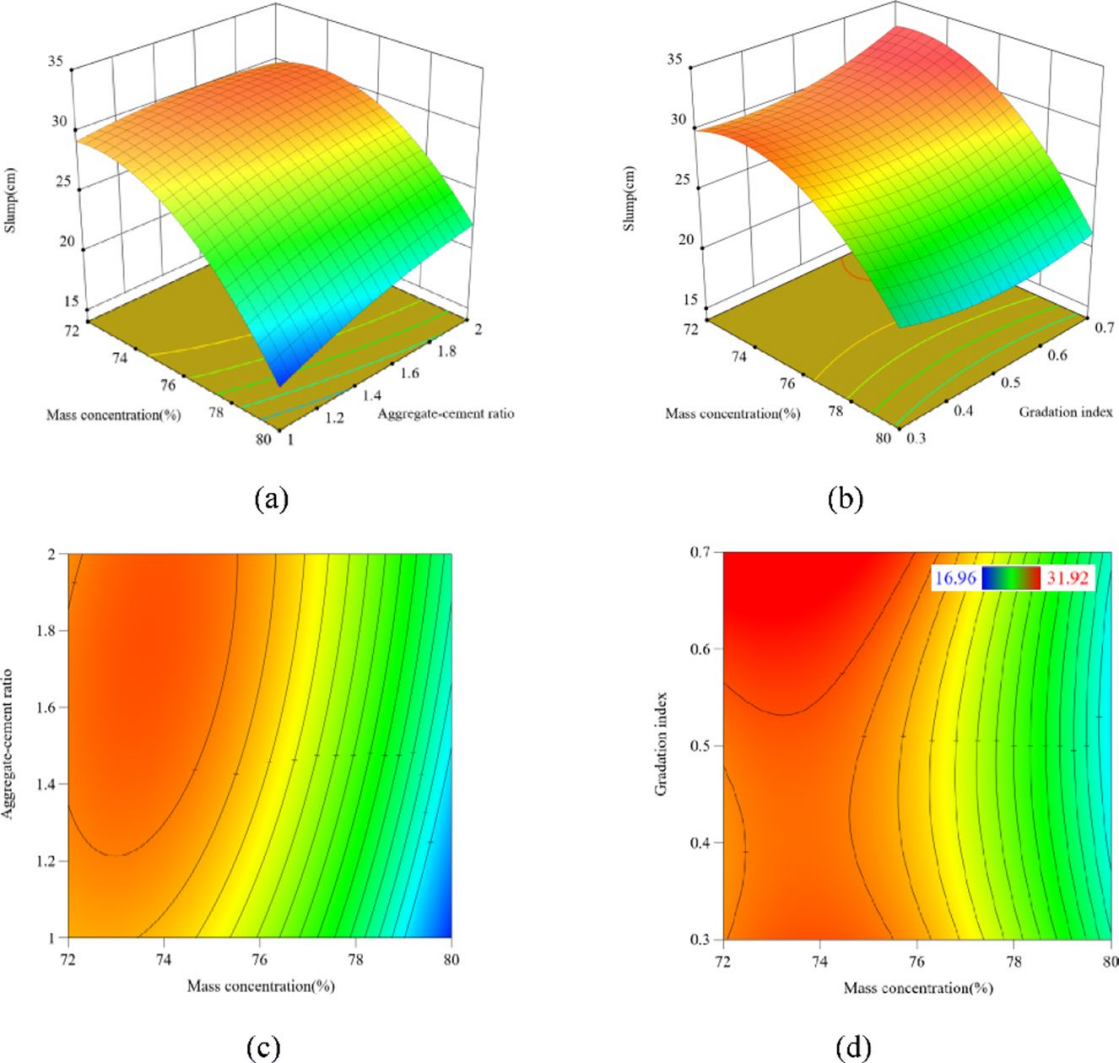
Response surface plots of slump: (**a**) and (**c**) mass concentration and aggregate-to-cement ratio; (**b**) and (**d**) mass concentration and grading index.

The interaction effects among the mass concentration, aggregate-to-cement ratio, and aggregate grading index upon the 3-, 14-, and 28-day UCSs of TSW-CPB were examined. As indicated in Table 2, the minimum P-values associated with the two-factor interaction terms in the UCS models at curing ages of 3 d, 14 d, and 28 d are separately 0.0108, 0.0003, and 0.1475. The most influential interaction term is (*X*_*1*_* × *_*2*_) (mass concentration × aggregate-to-cement ratio) for the 3-d and 14-d models, whereas for the 28-d model it shifts to (*X*_*1*_* × *_*3*_) (mass concentration × grading index). To visualize the relationship between factor interactions and the response, three-dimensional response surface plots were constructed by taking two factors (aggregate-to-cement ratio, mass concentration, or grading index) as the X/Y axes and UCS at each curing age as the Z axis (Figs. 12, 13, 14 and 15). In general, the curvature of response surfaces increases as the interaction effect becomes more significant, with elliptical contour lines indicating significant interactions, whereas near-circular contours imply weak or negligible interactions.

##### Response surface analysis for 3-d UCS

Figure 12 illustrates the effects of pairwise interactions on the 3-d UCS. With the grading index fixed at 0.5, the aggregate-to-cement ratio fixed at 1.5, and the mass concentration fixed at 76% (for the corresponding interaction pairs), all response surfaces are steep, and their curvature decreases progressively from Fig. 12(a) to Fig. 12(c).The associated contours are approximately elliptical. Among the interaction terms, (*X*_*1*_* × *_*2*_) has the smallest P-value (0.0108), confirming that the interaction between the aggregate-to-cement ratio and mass concentration is the most significant contributor to the 3-d strength, consistent with the response-surface features. As shown in Fig. 12(a), the 3-d UCS grows with increasing mass concentration and drops with increasing aggregate-to-cement ratio; moreover, with an increment in the ratio, the UCS becomes less sensitive to the mass concentration. The predicted maximum UCS is 0.652 MPa at (*X*_*1*_ = 80%) and (*X*_*2*_ = 1). Similar tendencies are also observed in Fig. 12(b) and Fig. 10(c); however, the interaction involving grading index is comparatively weak (e.g., (F = 0.0241 < 11.86)), indicating that the 3-d UCS is less sensitive to the grading index. Notably, the response surface in Fig. 12(c) opens slightly upward, suggesting that, under different grading indices, the 3-d UCS may exhibit a decrease–increase trend with increasing aggregate-to-cement ratio, and the interaction becomes more pronounced at lower aggregate-to-cement ratios—consistent with the main-effect results.

##### Response surface analysis for 14-d UCS

Figure 13 presents the pairwise interaction effects on the 14-d UCS, with the fixed levels under each interaction pair consistent with those in Fig. 12. The response surfaces remain steep, and the curvature is larger than that at the 3rd d; correspondingly, the F-values increase and the P-values decrease relative to the 3-d model (Table 2), indicating stronger interaction effects at the 14th d. As shown in Fig. 13(a), the contour lines become denser with a low mass concentration while a high aggregate-to-cement ratio, implying the more pronounced interaction in this region. At a low mass concentration, the UCS grows as the grading index increases; however, as mass concentration increases, the UCS tends to decrease with increasing grading index. Nevertheless, because mass concentration has a stronger main effect than grading index (as shown by the single-factor analysis), the overall trend remains an increase in UCS with increasing mass concentration, as also reflected in Fig. 13(b). Unlike the 3-d behavior, as the curing age increases, both the grading index and aggregate-to-cement ratio show negative correlations with the UCS within the investigated range, and the predicted maximum UCS (2.182 MPa) is obtained at (*X*_*2*_ = 1) and (*n* = 0.3).

##### Response surface analysis for 28-d UCS

Figure 14 depicts the pairwise interaction effects on the 28-d UCS. In Fig. 14(a), the surface is steep and forms a slightly downward-opening paraboloid. The contours are close to circular at a high aggregate-to-cement ratio while a low mass concentration; whereas they become increasingly elliptical when the mass concentration is high and the aggregate-to-cement ratio is low, indicating that increases in the solid particle content and binder fraction markedly enhance their interaction effect on the strength. The predicted maximum UCS is 5.533 MPa at (*X*_*1*_ = 80%) and (*X*_*2*_ = 1). Figure 14(b) shows that at relatively low mass concentration, the UCS increases with increasing grading index, whereas near (*X*_*1*_ = 80%), the UCS becomes negatively correlated with the grading index; the maximum is obtained at (*X*_*3*_ = 0.7) and (*X*_*1*_ = 80%). Figure 14(c) presents a relatively smooth inclined surface, indicating a weaker interaction; within the examined range, the UCS increases with the aggregate-to-cement ratio while decreases with the grading index, with its maximum located at (*X*_*2*_ = 1) and (*X*_*3*_ = 0.7).

##### Slump

Figure 15 summarizes the combined effect of pairwise interactions upon slump. Specifically, Fig. 15(a) evaluates the interaction between the mass concentration and the aggregate-to-cement ratio, showing an increment of the slump with the ratio while a reduction with the mass concentration. As the mass concentration rises, their interaction becomes more pronounced, indicating the strengthened regulating effect of the aggregate-to-cement ratio upon the slump under high-solids conditions. The maximum slump (31.92 cm) occurs at (*X*_*1*_ = 80%) and (*X*_*2*_ = 2), suggesting optimal flowability under this mix combination. Figure 15(b) further reveals the interaction between mass concentration and grading index. The corresponding response surface exhibits a typical saddle-shaped morphology, indicating a nonlinear synergistic effect. The gradient along the mass-concentration direction is more pronounced, confirming that mass concentration is one of the dominant factors governing slump, consistent with the single-factor analysis.

### Mechanism underlying strength evolution of the TSW-CPB

#### Hydration heat release characteristics

**Fig. 16:**
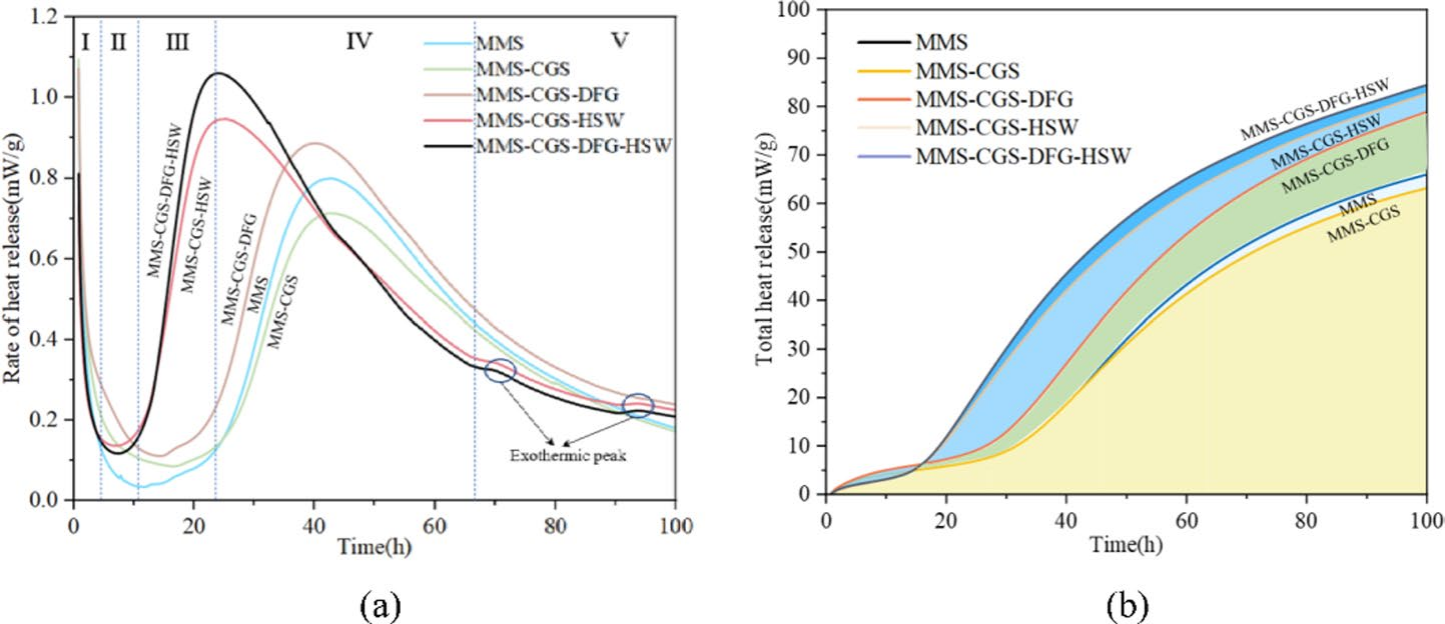
Cumulative heat release and heat-flow rate curves of the hydration reaction of TSW-CPB. (**a**) Cumulative heat release, (**b**) Heat-flow rate.

**Fig. 17:**
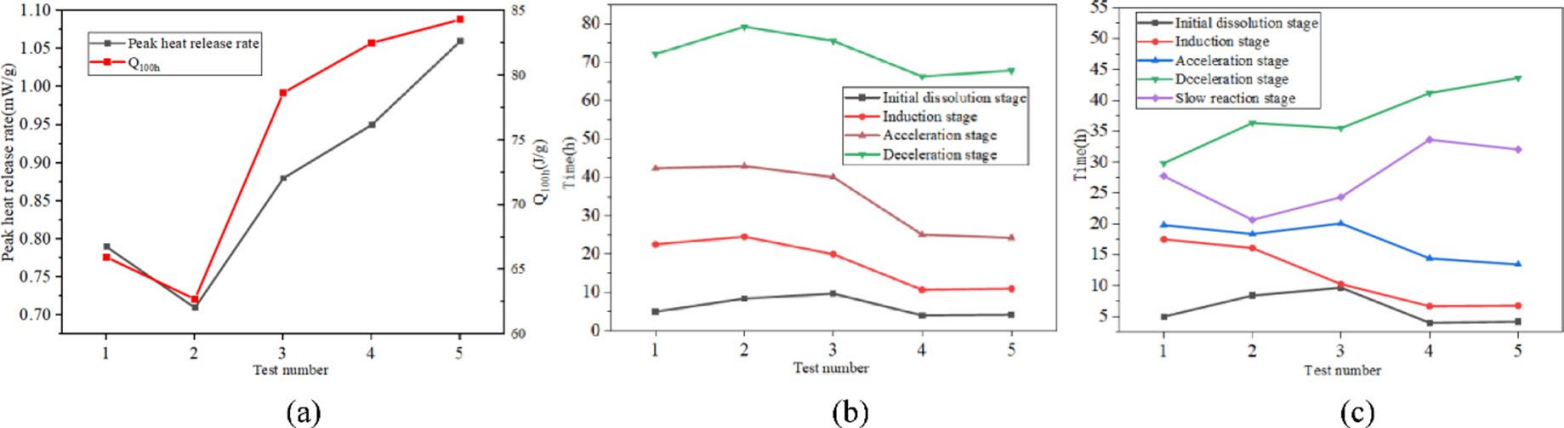
Hydration characteristics of TSW-CPB. (**a**) Cumulative heat release and peak heat-flow rate, (**b**) Onset time of each stage, (**c**) Duration of each stage.

To further elucidate the strength-giving phases of TSW-CPB and their evolution, hydration calorimetry was performed on five binder systems formulated from the four solid wastes, in order to clarify the hydration pathway of multi-component backfill materials based on solid waste. Figures 16 and 17 present the heat-flow rate (N) and cumulative heat release (Q) of TSW-CPB within 100 h. Similar to most cemented backfill binders, the hydration process falls into five stages: (I) the initial dissolution stage, (II) the induction stage, (III) the acceleration stage, (IV) the deceleration stage, and (V) the slow-reaction stage^37,47^. Within 100 h, relative to the MMS reference, the successive changes in cumulative heat release and peak heat-flow rate were (−3.23) (− 4.89%), 12.70 (19.25%), 16.55 (25.09%), and 18.36 (27.83%); and (−0.08) (− 10.12%), 0.09 (11.39%), 0.16 (25.25%), and 0.27 (34.18%) mW/g, respectively.

Using MMS as the baseline, incorporation of CGS into the binary binder retarded hydration, as evidenced by the delayed appearance of the secondary peak and the reduced peak intensity. This is primarily because the pozzolanic oxides in CGS require dissolution under alkaline conditions (via progressive attack) to release reactive Si/Al species for subsequent hydration, resulting in delayed onset and extended duration of the induction and acceleration stages, together with prolonged deceleration and slow-reaction stages, and thus a slight reduction in the overall heat release.

For the ternary binders, both MMS–CGS–DG and MMS–CGS–HSW exhibited markedly higher heat release, with the secondary-peak occurrence time falling between those of the unary and binary systems. Compared with MMS–CGS–DG, the MMS–CGS–HSW system showed a slightly earlier secondary peak, higher peak intensity, and a broader shoulder, indicating an accelerated hydration process. This enhancement is attributed to the participation of dissolved Ca^2+^ and SO2- 4 from DG in hydration reactions, while CGS mainly provides additional nucleation sites at early ages and is less reactive than MMS^48^. Meanwhile, Ca^2+^ and Cl^−^ in HSW facilitate C–A–S–H formation and reduce ionic activity of the pore solution, thereby noticeably shortening the induction stage while advancing the onset of the acceleration stage, which is beneficial for early-age strength development^49,50^. For the quaternary binder system, the onset times of all stages were the earliest, accompanied by the shortest durations of the acceleration stage and the slow-reaction stage, and the highest cumulative heat release. Moreover, DG and HSW jointly promoted hydration without mutual inhibition; instead, they exhibited a synergistic activation effect. This synergy was further interpreted mechanistically based on XRD results.

**Fig. 18:**
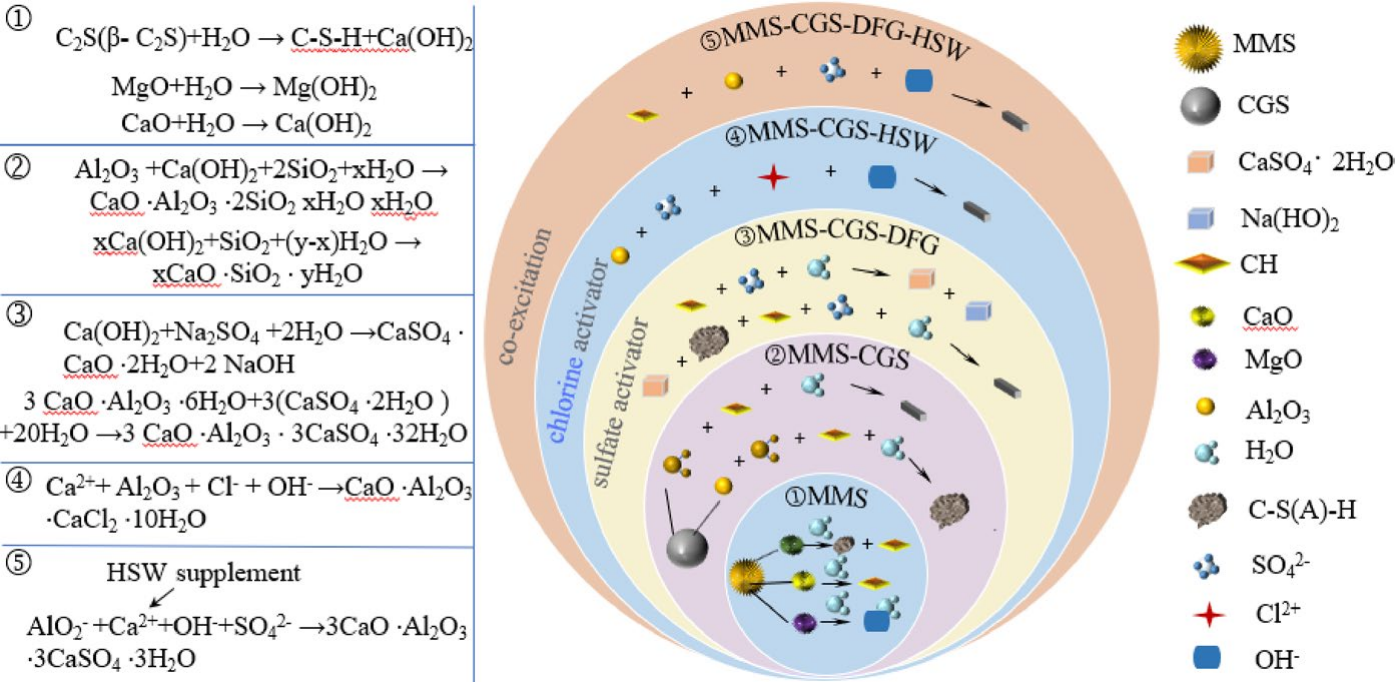
Schematic illustration of the hydration mechanisms of TSW-CPB.

The principal reactions occurring in the five solid-waste-based binder systems are schematically summarized in Fig. 18. When MMS is used as the unary binder, abundant β-C_2_S, MgO and CaO form Ca(OH)_2_, Mg(OH)_2_ and C–S–H by reacting with water. For the MMS–CGS binder, in the MMS-induced alkaline environment, reactive phases in CGS Al_2_O_3_ participate in hydration and yield products analogous to those in Portland-cement hydration; notably, the high OH^−^ concentration facilitates stable silicate/aluminate frameworks to depolymerize in CGS, promoting breakage of Si–O–Al and Si–O–Si bonds and enabling more $\:{\mathrm{A}\mathrm{l}\mathrm{O}}_{2}^{-}$ to enter the solution or react at the CGS surface. In the MMS–CGS–DG system, DG readily dissolves and can react with CH to form CaSO_4_·2H_2_O, which further reacts with calcium aluminate hydrates (C–A–H) to produce AFt; meanwhile, the alkalinity of the pore solution is enhanced, providing a more favorable hydration environment. In the MMS–CGS–HSW system, HSW can form Friedel’s salt by reacting with AFt; an appropriate amount of the salt contributes to pore filling and strength enhancement^51,52^. In the quaternary MMS–CGS–DG–HSW binder, SO2- 4may exchange with Cl^−^ in Friedel’s salt, ultimately favoring AFt formation^53^.

#### Microstructural evolution

**Fig. 19:**
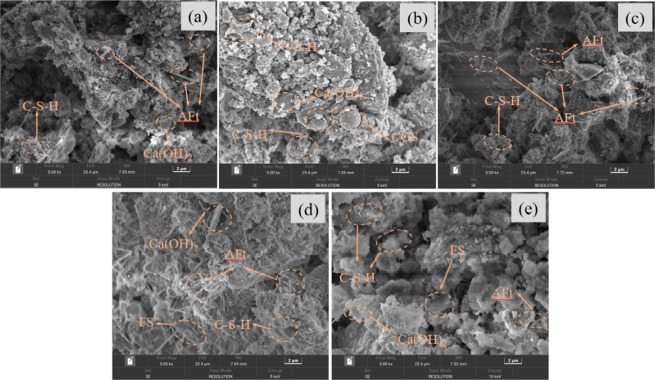
SEM micrographs of TSW-CPB at the 28th day.

Figure 19 shows the SEM micrographs of specimens with different binder systems at the 28th day. For the unary binder MMS (Fig. 19a), remnants of unreacted CGS particles can be identified, together with a widely distributed flocculent network of C–S(A)–H gel, consistent with the XRD results and the hydration-heat-based mechanistic interpretation. With the incorporation of CGS (Fig. 19b), spherical vitreous CGS particles and a small fraction of angular CGS fragments are observed, embedded and coated by flocculent C–S(A)–H and needle/rod-like AFt (ettringite)^54^. This microstructure indicates that aluminosilicate phases within CGS participate in hydration under the MMS-induced alkaline environment. Besides, a limited amount of CH is detected, suggesting that contribution of CGS to strength in the early stage is largely associated with physical filling rather than extensive chemical reaction, because CGS exhibits lower reactivity than MMS.

**Fig. 20:**
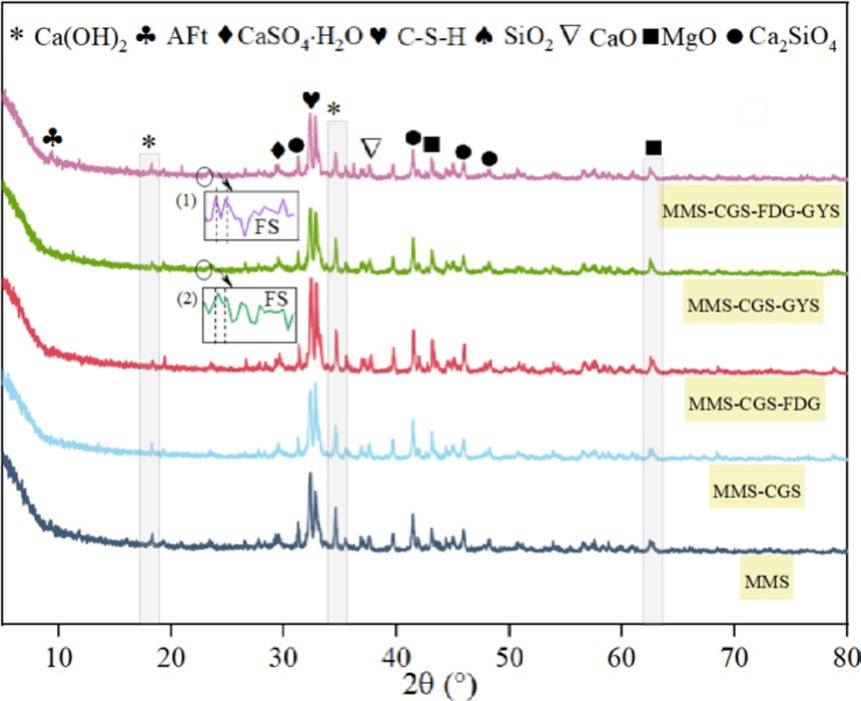
XRD patterns of TSW-CPB at the 28th day.

After adding DG (Fig. 19c), the hexagonal plate-like CH crystals observed in Figs. 19(a) and (b) are no longer evident. This is mainly because DG promotes pozzolanic reactions of reactive components in the system, thereby consuming CH. Meanwhile, compared with Fig. 19, more columnar AFt with a coarser morphology and denser distribution is developed^47^. In Figs. 19(d) and (e), thin hexagonal Friedel’s salt (FS) plates—distinguishable from CH by their smaller thickness—are observed, together with an interwoven assemblage of AFt hydration products and C–S–H gel. This observation is further confirmed by XRD patterns presented in Fig. 20. The diffraction spectra clearly reveal distinct phase compositions between the two systems. In the MMS–CGS–HSW system, the characteristic peaks corresponding to ettringite (AFt) are particularly prominent, especially in the low-angle region, indicating a higher abundance of this phase. In contrast, the XRD pattern of the MMS–CGS–DG–HSW system is dominated by a broad amorphous hump in the range of 30–38° 2θ, a typical feature of C–S–H gel, while peaks associated with AFt are noticeably attenuated. The marked difference in the dominant phases—where AFt predominates in the former system while the C–S–H gel serves as the primary hydration product of the latter—provides direct microstructural evidence for the observed divergence in the macroscopic properties of the two formulations.

### MOO based on NSGA-III

#### MOO based on NSGA-III

Because most mines adopting concurrent mining and backfilling (backfill-as-you-mine) commonly implement layered backfilling, the hardened backfill in the lower layer serves as the working platform for subsequent filling in the upper layer. Therefore, the rate of early-age strength development directly governs mining–backfilling coordination. Accordingly, the 3-, 14-, and 28-day UCSs were selected as the strength-related optimization objectives in this study, together with an economic indicator based on raw-material purchase costs (CGS: 35 ¥/ton); MMS: 40 ¥/ton; DG: 30 ¥/ton; coal gangue: 5 ¥/ton). The constraints were defined according to slurry workability: mixtures with a slump of 15–20 cm require pumping, whereas those with a slump of 23–27 cm are suitable for self-flowing placement; optimization was thus performed separately for the two placement modes. Consequently, the MOO model for TSW-CPB performance and the corresponding constraints on the decision variables can be expressed as Eqs. (14) and (15).

14 $$\left\{ {\begin{array}{*{20}{c}} {\hbox{max} (3dUCS)={f_1}{{({x_{1,}}{x_{2,}}{x_3})}_{}}} \\ {\hbox{max} (14dUCS)={f_2}({x_{1,}}{x_{2,}}{x_3})} \\ {\hbox{max} (28dUCS)={f_3}({x_{1,}}{x_{2,}}{x_3})} \\ {Min(\operatorname{Cos} t)={f_4}({x_{1,}}{x_{2,}}{x_3})} \end{array}} \right.$$

15 $$s.t.\left\{ {\begin{array}{*{20}{c}} {0.76 \leqslant {x_1} \leqslant 0.80} \\ {1 \leqslant {x_1} \leqslant 2} \\ {0.3 \leqslant {x_1} \leqslant 0.7} \\ {15 \leqslant {Y_4} \leqslant 20(Scenario1:Puping)} \\ {23 \leqslant {Y_4} \leqslant 27(Scenario2:Self - flowing)} \end{array}} \right.$$

#### Obtainment of the Pareto front

MOO was carried out on the basis of the defined constraints and objective functions, with crossover and mutation parameters separately set to 100 and 0.5. Specifically, the population size and the Pareto set fraction were set to be 100 and 0.5, respectively. For the selection operator, tournament selection was used. An intermediate crossover operator and an adaptive feasible mutation operator were employed, with the crossover fraction and mutation fraction separately being 0.9 and 0.1. The termination criterion for NSGA-III was a maximum of 2000 generations in this study.

**Fig. 21:**
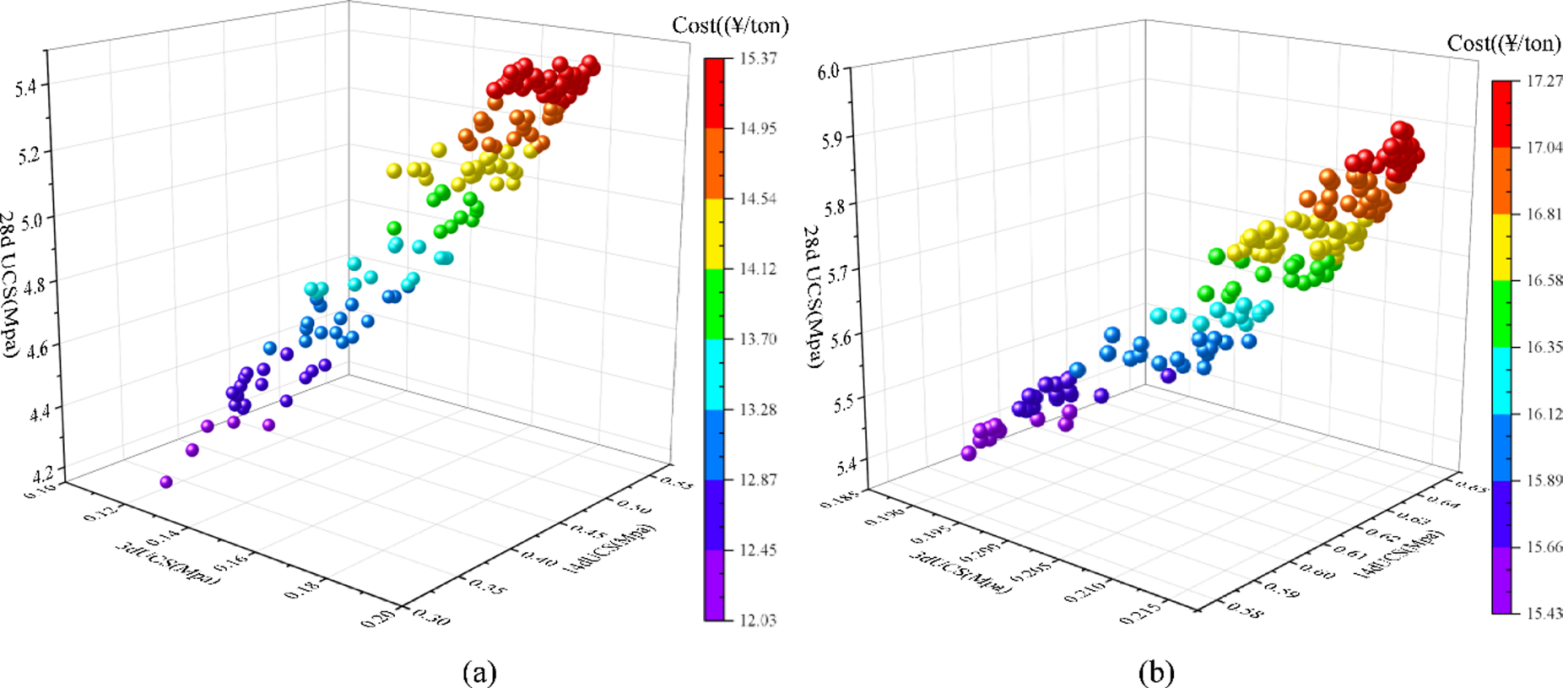
Pareto front. (**a**) Pumping, (**b**) Self-flowing.

In addition, the Pareto front was partitioned into three segments according to slump: mixtures with a slump of 15–20 cm are suitable for pumped transport, whereas those with a slump of 23–27 cm can be placed under self-flowing conditions. Figure 21 shows the approximate planar distribution of the Pareto front, indicating satisfactory convergence^55^. Moreover, strength and workability exhibit a negative correlation; therefore, simultaneous improvement in both flowability and strength is generally unattainable, and the optimal solution should be selected according to specific engineering requirements.

### Determination of the optimal mix proportion using the entropy-based TOPSIS approach

#### Distribution of weights of key performance indicators

Based on the Pareto-optimum solution attained from NSGA-III, the entropy-weighting method was further applied according to Eqs. (3)–(11) to determine the objective-weight coefficients for the 3-, 14-, and 28-day UCSs and the raw-material purchase cost under pumping and self-flowing conditions. The resulting weights were 0.3054, 0.2841, 0.3047, and 0.1059 for the pumping mode, and 0.2751, 0.3739, 0.2126, and 0.1384 for the self-flowing mode, respectively.

**Table 4:** Weight coefficient of each factor.

Factor	Entropy value		Coefficient of difference		Weight	
	Pumping	Self-flowing	Pumping	Self-flowing	Pumping	Self-flowing
3-d UCS	0.9365	0.9519	0.0635	0.0481	0.3054	0.2751
14-d UCS	0.9409	0.9347	0.0591	0.0653	0.2841	0.3739
28-d UCS	0.9367	0.9629	0.0633	0.0371	0.3047	0.2126
Raw material purchase cost	0.9780	0.9758	0.0219	0.0247	0.1059	0.1384

#### Decision on the optimal solution and verification

According to the closeness coefficient (*C*_*i*_), Pareto-optimum solutions were ranked utilizing the TOPSIS method. Under the two workability constraints (pumping and self-flowing), the top five optimal solutions for each operating condition were identified, as summarized in Tables 4, 5. In the optimal mix proportion for the pumping condition, the mass concentration, aggregate-to-cement ratio, and aggregate grading index are separately 78.758%, 1.3911, and 0.30504. For the self-flowing condition, the optimal mix proportion is as follows: the mass concentration, aggregate-to-cement ratio, and aggregate grading index are separately 77.527%, 1.0003, and 0.5864.

**Table 5:** Results of the TOPSIS method based on entropy.

NO.	Mass concentration (%)		Aggregate-to-cement ratio		Grading index		3-d UCS (MPa)		14-d UCS (MPa)		28-d UCS (MPa)		Cost/(¥/ton)	
Category	Pumping	Self-flowing	Pumping	Self-flowing	Pumping	Self-flowing	Pumping	Self-flowing	Pumping	Self-flowing	Pumping	Self-flowing	Pumping	Self-flowing
1	0.78758	0.77527	1.3911	1.0003	0.30504	0.58684	0.1791	0.2132	0.5423	0.6477	5.353	5.82	15.15	17.06
2	0.79163	0.77222	1.3989	1	0.3063	0.5692	0.1793	0.2125	0.5408	0.6486	5.368	5.805	15.19	17
3	0.79318	0.77706	1.3994	1.0002	0.30401	0.59698	0.1797	0.2137	0.5415	0.6473	5.377	5.841	15.21	17.1
4	0.79744	0.77957	1.3918	1	0.30071	0.60739	0.1814	0.2144	05462	0.6473	5.414	5.829	15.33	17.16
5	0.78458	0.77706	1.3982	1.0003	0.31891	0.60172	0.1776	0.2137	0.5366	0.6467	5.327	5.842	15.05	17.1

## Conclusions

(1) Response surface models for the 3-, 14-, and 28-day UCSs and for slump were developed using an RSM–BBD within the factor space including the mass concentration (72–80%), aggregate-to-cement ratio (1–2), and aggregate grading index (0.3–0.7). ANOVA results indicate that all models show high significance (*P* < 0.0001) without significant lack-of-fit (*P* > 0.05), demonstrating their applicability for mix prediction and subsequent multi-objective optimization.

(2) Within the investigated ranges, the UCS increases with the mass concentration while decreases with aggregate-to-cement ratio. Effects of the grading index on both strength and workability are condition-dependent, highlighting an inherent trade-off between strength and flowability that necessitates multi-objective coordination.

(3) Under the slump constraints (15–20 cm for pumping; 23–27 cm for self-flowing), NSGA-III generated a Pareto-optimum solution set. Coupling with entropy-weighted TOPSIS enabled quantitative ranking and screening within different workability intervals, improving the interpretability and engineering practicability of the recommended mix designs.

(4) The optimal mix proportions were identified for the two placement modes: for pumping, the mass concentration, aggregate-to-cement ratio, and grading index are 78.758%, 1.3911, and 0.30504, respectively; for self-flowing, they are 77.527%, 1.0003, and 0.5864, respectively. The above parameterized mix proportions provide direct guidance for zoned backfilling practice under pumping/self-flowing conditions.

(5) Mechanistic characterization indicates that incorporating CGS retards hydration in the binary system, whereas DG and HSW markedly promote heat evolution and hydration product formation. In particular, HSW induces Friedel’s salt formation and pore filling, which enhances matrix densification and supports sustained strength development and system stabilization.

## Data Availability

The datasets used and/or analysed during the current study available from the corresponding author on reasonable request.
